# Generation of complex bone marrow organoids from human induced pluripotent stem cells

**DOI:** 10.1038/s41592-024-02172-2

**Published:** 2024-02-19

**Authors:** Stephanie Frenz-Wiessner, Savannah D. Fairley, Maximilian Buser, Isabel Goek, Kirill Salewskij, Gustav Jonsson, David Illig, Benedicta zu Putlitz, Daniel Petersheim, Yue Li, Pin-Hsuan Chen, Martina Kalauz, Raffaele Conca, Michael Sterr, Johanna Geuder, Yoko Mizoguchi, Remco T. A. Megens, Monika I. Linder, Daniel Kotlarz, Martina Rudelius, Josef M. Penninger, Carsten Marr, Christoph Klein

**Affiliations:** 1 Department of Pediatrics, Dr. von Hauner Children’s Hospital, University Hospital, Ludwig-Maximilians-University Munich, Munich, Germany; 2 Institute of Cardiovascular Prevention (IPEK), Ludwig-Maximilians-University Munich, Munich, Germany; 3 Institute of AI for Health, Helmholtz Zentrum München—German Research Center for Environmental Health, Neuherberg, Germany; 4 Institute of Molecular Biotechnology of the Austrian Academy of Sciences (IMBA), Vienna BioCenter (VBC), Vienna, Austria; 5 Vienna BioCenter PhD Program, Doctoral School of the University of Vienna and Medical University of Vienna, Vienna, Austria; 6 Institute of Diabetes and Regeneration Research, Helmholtz Diabetes Center, Helmholtz Center Munich, Neuherberg, Germany; 7 German Center for Diabetes Research (DZD), Neuherberg, Germany; 8 Technical University of Munich, Munich, Germany; 9 Anthropology and Human Genomics, Faculty of Biology, Ludwig-Maximilians-University Munich, Martinsried, Germany; 10 Department of Pediatrics, Graduate School of Biomedical Sciences, Hiroshima University, Hiroshima, Japan; 11 Department of Biomedical Engineering (BME), Cardiovascular Research Institute Maastricht (CARIM), Maastricht University Medical Centre, Maastricht, The Netherlands; 12 German Center for Cardiovascular Research (DZHK), Partner Site Munich Heart Alliance, Munich, Germany; 13 Institute of Pathology, Faculty of Medicine, Ludwig-Maximilians-University Munich, Munich, Germany; 14 Department of Laboratory Medicine, Medical University of Vienna, Vienna, Austria; 15 Helmholtz Centre for Infection Research, Braunschweig, Germany; 16 Department of Medical Genetics, Life Sciences Institute, University of British Columbia, Vancouver, Canada; 17 Gene Center, Ludwig-Maximilians-University Munich, Munich, Germany

**Keywords:** Haematopoietic stem cells, Haematopoiesis, Stem-cell niche, Biological models

## Abstract

The human bone marrow (BM) niche sustains hematopoiesis throughout life. We present a method for generating complex BM-like organoids (BMOs) from human induced pluripotent stem cells (iPSCs). BMOs consist of key cell types that self-organize into spatially defined three-dimensional structures mimicking cellular, structural and molecular characteristics of the hematopoietic microenvironment. Functional properties of BMOs include the presence of an in vivo-like vascular network, the presence of multipotent mesenchymal stem/progenitor cells, the support of neutrophil differentiation and responsiveness to inflammatory stimuli. Single-cell RNA sequencing revealed a heterocellular composition including the presence of a hematopoietic stem/progenitor (HSPC) cluster expressing genes of fetal HSCs. BMO-derived HSPCs also exhibited lymphoid potential and a subset demonstrated transient engraftment potential upon xenotransplantation in mice. We show that the BMOs could enable the modeling of hematopoietic developmental aspects and inborn errors of hematopoiesis, as shown for human VPS45 deficiency. Thus, iPSC-derived BMOs serve as a physiologically relevant in vitro model of the human BM microenvironment to study hematopoietic development and BM diseases.

## Abstract (web summary)

The authors describe stem cell-derived bone marrow organoids that accurately model the structural and functional properties of the human bone marrow niche.

## Main

Human hematopoiesis in the BM is a tightly regulated process permitting continuous differentiation of hematopoietic stem cells (HSCs) into mature blood cells, while maintaining an HSC pool through self-renewal^1^. Vascular and mesenchymal cells constitute the surrounding BM niche regulating hematopoiesis throughout life^2^. The processes of vasculogenesis, osteogenesis and, subsequently, hematopoiesis are tightly coupled in the fetal BM^3^. Genetic mutations of BM niche cells have been shown to be sufficient to induce myelodysplasia or leukemia^4,5^, emphasizing their critical role not only in development, but also in disease evolution. Murine models of the BM niche have been widely studied^4–7^, but are limited due to species-specific differences in hematopoiesis^8^. Recently, methods for generating induced pluripotent stem cell (iPSC)-derived hematopoietic organoids^9^ and a first BMO model^10^ have been developed. Here, we report an advanced in vitro model of the human BM microenvironment that can recapitulate key functional and structural features of the human BM hematopoietic niche with high fidelity.

## Results

### Development of human BMOs

**Fig. 1:**
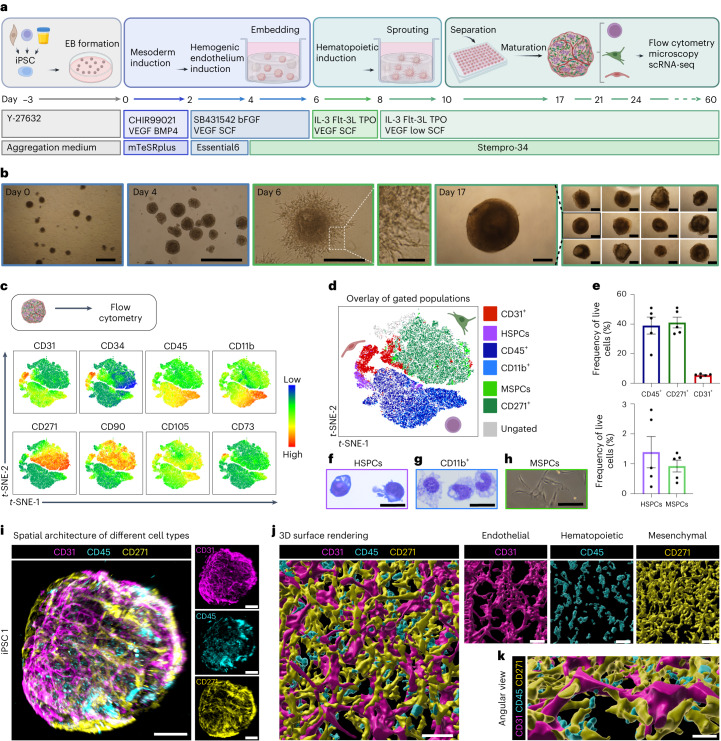
Generation of human iPSC-derived BMOs and analysis of cellular composition by flow cytometry and microscopy. **a**, Schematic illustration of the workflow for BMO generation. **b**, Representative bright-field images of iPSC1-derived embryoid bodies on day 0 and day 4, sprouting embryoid bodies on day 6 and differentiated BMOs on day 17 of *n* = 5 independent differentiations. **c**, Joint *t-*SNE visualization of day 17 flow cytometric data of *n* = 3 independent differentiations resulting in 105,000 cells. Expression of each lineage marker is shown in the *t-*SNE plots. Color correlates with intensity of expression (red, high expression; blue, low expression). **d**, Overlay of manually gated populations on *t-*SNE projection. Subsets defined as endothelial (CD45^−^CD31^+^), hematopoietic (CD45^+^), myeloid (CD45^+^CD11b^+^) and mesenchymal (CD45^−^CD31^−^CD271^+^) cells. HSPCs as CD45^+^CD11b^−^CD34^+^ and MSPCs as CD45^−^CD31^−^CD271^+^CD90^+^CD105^+^CD73^+^. Detailed gating strategy is outlined in Extended Data Fig. 1a. **e**, Quantification of frequencies of manually gated cell types for *n* = 5 independent differentiations. Data are presented as mean values ± s.e.m. **f**,**g**, Representative bright-field microscopy of sorted and May–Gruenwald–Giemsa-stained HSPCs (**f**) and CD45^+^CD11b^+^ myeloid cells (**g**) of *n* = 3 independent differentiations. Characteristic high nuclear-to-cytoplasm ratio of HSPCs. CD11b^+^ myelomonocytic cells, including monocytic cells (bean-shaped nucleus, a pale cytoplasm and fine granules) and immature neutrophil granulocyte characterized by the band-shaped nucleus. **h**, Morphology of sorted MSPCs in culture by phase-contrast. Representative image of *n* = 6 experiments with iPSC1 and iPSC2. **i**, Analysis of a representative whole-mount organoid (iPSC1, day 21) stained with indicated antibodies using two-photon microscopy of *n* = 3 independent differentiations. **j**, Surface rendering of a 3D *z*-reconstruction from a confocal image displaying CD45^+^ cells in CD31^+^ and CD271^+^ niche cells. **k**, Still image from Supplementary Video 5. Angular view of **j** to visualize 3D architecture. Scale bars, **b**, 500 µm (day 6 inset 100 µm); **f**–**h**, 20 µm; **i**, 100 µm; **j**,**k**, 50 µm. Source data

**Extended Data Fig. 1:**
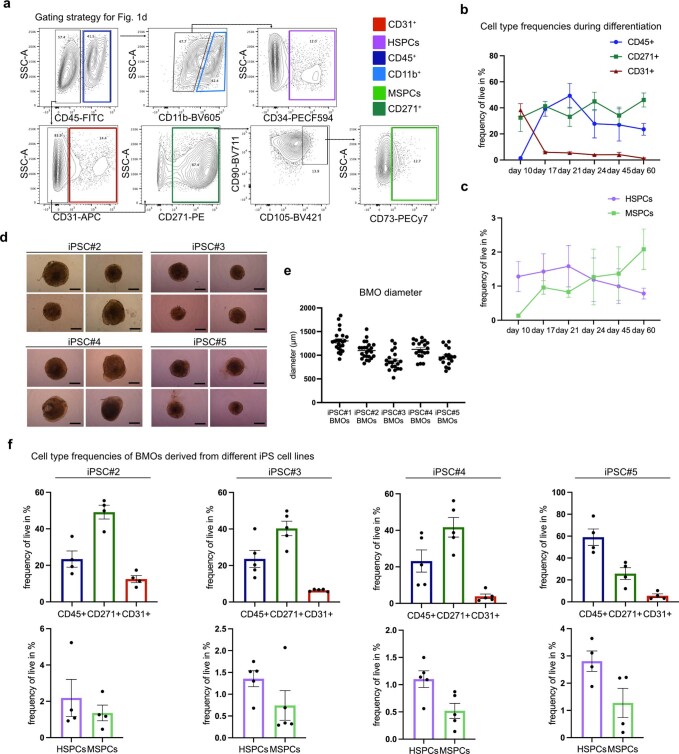
Flow cytometry analysis, morphology and cell type frequencies of BMOs across various iPSC lines. **a)** Gating strategy for flow cytometric analysis of dissociated BMOs. **b)** Quantification of cell type frequencies for main fractions of iPSC1-BMOs day 10-60; *n* = 5 independent differentiations for day 17, 21, *n* = 3 independent differentiations for day 10, 24, 45, 60. **c)** Quantification of cell type frequencies of HSPCs and MSPCs of iPSC1-BMOs day 10-60; *n* = 5 independent differentiations for day 17, 21, *n* = 3 independent differentiations for day 10, 24, 45, 60. **d)** Bright field images of BMOs generated from iPSC2-5 at day 17. **e)** Mean diameter of iPSC1-5-derived BMOs on day 17 of differentiation for *n* = 24 (iPSC1 and 2), *n* = 20 (iPSC3 and 4) and *n* = 17 (iPSC5) organoids. Bar indicates mean value. **f)** Quantification of cell type frequencies of iPSC2-5-BMOs on day 17, iPSC2 *n* = 5 independent differentiations; iPSC3, 4, 5 *n* = 3 independent differentiations. Data in **b), c)** and **f)** are presented as mean values ± s.e.m. Scale bar in d) 500µm. Source data

We devised a feeder- and serum-free protocol of stepwise mesodermal progenitor differentiation through hemogenic endothelium from iPSCs^11–15^ to generate a complex organoid consisting of hematopoietic, mesenchymal and endothelial cells (ECs) within 3 weeks (Fig. 1a). After embryoid body (EB) formation (day −3), mesoderm was induced by the Wnt agonist CHIR99021, bone morphogenetic protein 4 (BMP4) and vascular endothelial growth factor (VEGF) (day 0). Subsequently, mesoderm patterning and hemogenic endothelium (HE) induction was achieved using the activin/nodal pathway inhibitor SB431542, basic fibroblast growth factor (bFGF), stem cell factor (SCF) and VEGF (day 2). To promote organoid self-assembly, we adapted the embedding method of iPSC-derived blood vessel organoids in a collagen I/matrigel matrix^16,17^. Patterned EBs were embedded into this matrix on day 4 and stimulated with defined cytokines to generate hematopoietic progenitor cells while maintaining EC generation (Fig. 1a). To enhance vascular structure formation^16,17^, we added a low dose of VEGF from day 8 onwards. On day 10 of differentiation, sprouted EBs (Fig. 1b) were separated and transferred into single wells of an ultra-low-attachment 96-well plate to promote organoid maturation. To evaluate intrinsic emergence of mature lineages, we did not include lineage-directing cytokines. On day 17, the organoids assembled into a spherical shape (Fig. 1b) with a mean diameter of 1,300 µm (range 1,208–1,396 µm, 95% confidence interval (CI) of mean; Extended Data Fig. 1e).

### Heterogenous cell type composition

To examine the presence of essential cell types of human BM, we analyzed dissociated BMOs at day 17 of differentiation by flow cytometry (Fig. 1c). To visualize subset heterogeneity, we performed dimensionality reduction of flow cytometric data by *t*-distributed stochastic neighbor embedding (*t-*SNE)^18^ (Fig. 1c). We integrated data from three independent differentiations into a joint *t-*SNE visualization, generating a representative map across experiments (Fig. 1c and Supplementary Fig. 1b). Overlay of manually gated populations (Extended Data Fig. 1a and Supplementary Fig. 1a) revealed three main cell clusters (Fig. 1d). Based on CD45 as a panleukocyte marker, CD31 (PECAM1) as a marker for vascular ECs and CD271 (NGFR) as marker for mesenchymal cells, these clusters were assigned as endothelial (CD45^−^CD31^+^), hematopoietic (CD45^+^) and mesenchymal stromal cell clusters (CD45^−^CD31^−^CD271^+^), respectively. Hematopoietic cells were further defined as HSPCs (CD45^+^CD11b^−^CD34^+^) and myeloid cells (CD45^+^CD11b^+^). CD45^−^CD31^−^CD271^+^CD90^+^CD105^+^CD73^+^ cells were characterized as mesenchymal stem/progenitor cells (MSPCs) according to the minimal criteria for multipotent MSPCs^19^ (Fig. 1d and Extended Data Fig. 1a). Quantitative analysis of the cellular composition on day 17 of culture yielded an average content of 39.3% hematopoietic cells, 41.3% mesenchymal cells, 6.0% ECs, 1.42% HSPCs and 0.96% MSPCs per BMO (*n* = 5; Fig. 1e). This relative cell type composition was constant at days 17–24 of differentiation (Extended Data Fig. 1b,c). Although each subset was still detectable up to day 60 of differentiation, the frequency of CD45^+^ cells declined over time (Extended Data Fig. 1b,c and Supplementary Fig. 1d–g). (See Supplementary Note and Supplementary Fig. 2 describing our strategies to determine reproducibility of the protocol using iPSC lines 2–5.) To validate the immunophenotypic subsets morphologically, we sorted distinct subsets using fluorescence-activated cell sorting (FACS) (Fig. 1f,g,h). Giemsa-stained cytospins of isolated HSPCs (CD45^+^CD11b^−^CD34^+^) and myeloid cells (CD45^+^CD11b^+^) showed characteristic morphology of myeloid progenitor cells (Fig. 1f) and monocytoid and granulocytic cells (Fig. 1g), respectively. Sorted BMO-derived MSPCs (CD45^−^CD31^−^CD90^+^CD105^+^CD271^+^CD73^+^) were adherent to plastic and possessed spindle-like morphology (Fig. 1h), characteristic of MSPCs^19^. Moreover, these cells expanded in culture and had serial replating capacity for up to 15 passages.

**Extended Data Fig. 2:**
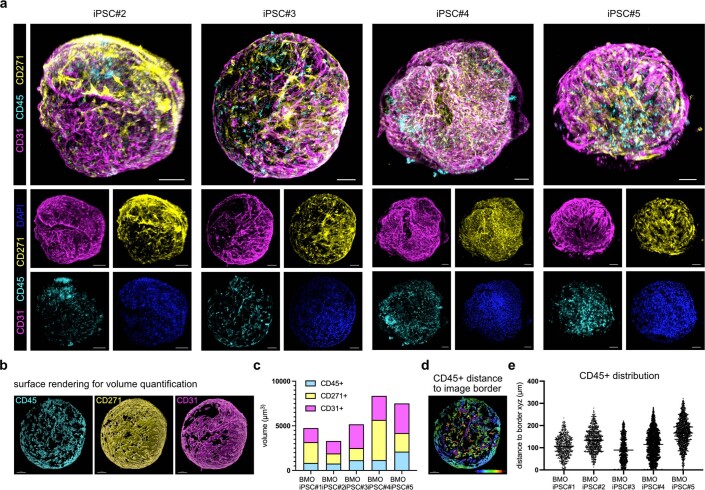
Complex BMO architecture across various iPS cell lines. **a)** Analysis of representative mature whole-mount organoid (iPSC2-5) stained with indicated antibodies using two-photon microscopy; *n* = 3 independent experiments. **b)** Representative surface rendering of CD45, CD271 and CD31 expressing cells for volume quantification of *n* = 5 organoids (iPSC1-5). **c)** Quantification of indicated cell types within BMOs (*n* = 5 organoids) derived from iPSC1-5. **d)** Coloring of CD45+ cells based on distance to image border. **e)** Quantification of the spatial CD45+ distribution as measured by distance to image border in XYZ dimension for *n* = 647 (iPSC1), 822 (iPSC2), 1119 (iPSC3), 2761 (iPSC4) and 1494 (iPSC5) CD45+ surface objects. Scale bars in a), b), d) 100µm. Source data

Next, we analyzed the spatial organization of the three main cell types by fluorescence microscopy. To increase the depth of imaging, we applied two-photon microscopy, allowing us to capture fluorescent signals of up to 845 µm depth. These studies confirmed the presence of vascular, hematopoietic and mesenchymal structures throughout the whole organoid (Fig. 1i and Supplementary Video 1). Confocal microscopy revealed spherical shaped CD45^+^ hematopoietic cells embedded into a network of CD31^+^ vascular structures and CD271^+^ stromal cells, which were visualized by three-dimensional (3D) surface rendering (Fig. 1j,k and Supplementary Fig. 3). Importantly, the spatial architecture of BMOs generated from different iPS cell lines was comparable (Extended Data Fig. 2a–c). Moreover, we examined the spatial localization of CD45^+^ hematopoietic cells in the BMO by 3D surface rendering and subsequent statistical analysis, indicating their distribution throughout the organoid (Extended Data Fig. 2d,e and Supplementary Video 2). Thus, our protocol enables the generation of BMOs derived from various iPS cell lines and donor backgrounds. BMOs self-organized into spatially defined 3D structures and consisted of key cell types characteristic of the human BM niche.

### Mesenchymal and vascular spatial architecture

**Fig. 2:**
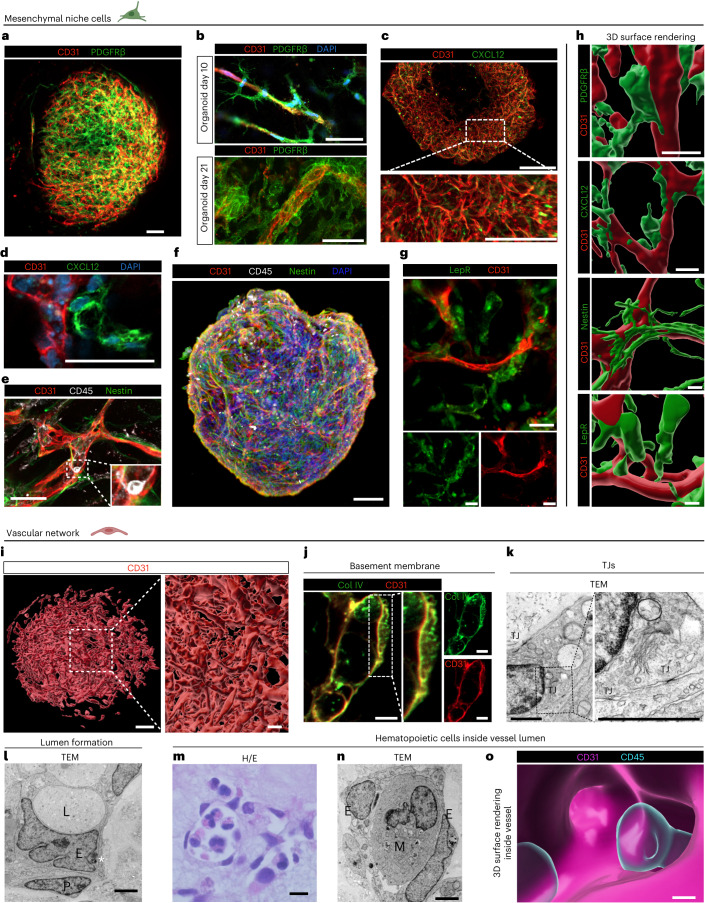
Spatial architecture of BMOs recapitulates key features of mesenchymal and vascular human BM niche. **a**, Confocal images of immunostaining of CD31 (ECs) and PDGFRβ (pericytes) in a BMO to visualize vascular network and PDGFRβ^+^ pericytes. **b**, Analysis of pericyte–EC association on day 10 and day 21 of differentiation using confocal microscopy. Top, finger-like extensions of PDGFRβ^+^ pericytes on day 10 of differentiation. Bottom, tight association of a PDGFRβ^+^ pericyte with an EC on day 21 of differentiation. **c**, Analysis of CXCL12 expression in specific cell subsets throughout the organoid visualized by confocal microscopy. **d**, Analysis of CXCL12-expressing pericytes in close association to ECs by confocal microscopy. **e**, Nestin^+^ cells lining CD31^+^ vessels and CD45^+^ hematopoietic cells. Note CD45^high^-expressing cell with banded nucleus. **f**, Analysis of Nestin expression in spatial association with CD31^+^ vessels and CD45^+^ hematopoietic cells by two-photon microscopy; *z*-dimension, 515 µm. **g**, Analysis of LepR expression by confocal microscopy. In **a**–**g**, *n* = 3 independent experiments. **h**, 3D surface rendering of mesenchymal niche cells and vessels. **i**, 3D surface rendering of optically cleared whole organoid stained for CD31; still frame from Supplementary Video 4. **j**, ECs are covered by a Col IV^+^ basement membrane. In **i** and **j**, *n* = 3 independent experiments. **k**, Analysis of a pericyte and EC connected by tight junctions (TJ) by TEM. **l**, TEM analysis of an organoid section showing capillary-like structure by an EC (E) forming a lumen (L) and enclosed by a pericyte (P); asterisk, Weibel–Palade bodies. **k** and **l** are representative micrographs of sections from *n* = 2 independent experiments. **m**, Histological sections reveal morphologically hematopoietic cells in lumen of vessel-like structures by H/E stain; *n* = 3 independent experiments. **n**, TEM analysis of an organoid section showing ECs (E) encompassing round cells resembling myeloid cells (M). Representative micrographs of sections from *n* = 2 independent experiments. **o**, Hematopoietic cells inside vessel lumen visualized by 3D rendering; still frame from Supplementary Video 5. Scale bars, **a**, **f**, **i** 100 µm, inset in **i** 50 µm; **b**–**e**, 50 µm; **g**, **m**, 20 µm; **h**, **j**, 10 µm; **k**, 1 µm; **l**, **n**, 2 µm; **o**, 5 µm.

**Extended Data Fig. 3:**
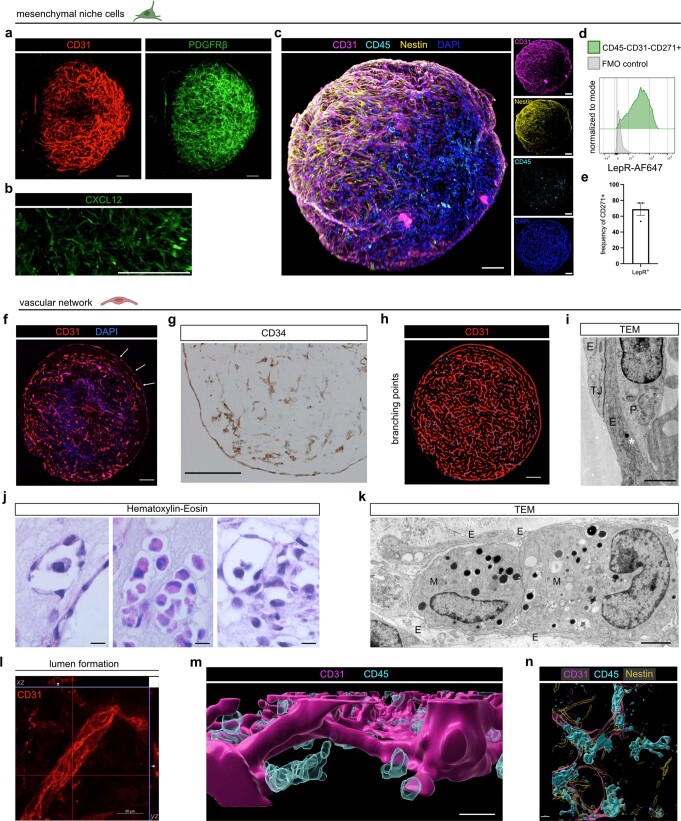
Characterization of mesenchymal and vascular structures. **a)** Confocal images of immunostaining of CD31 (endothelial cells) and PDGFRβ (pericytes) in a BMO; related to Fig. 2a. **b)** Analysis of CXCL12-expressing pericytes by confocal microscopy as shown in merged image in Fig. 2e. **c)** Analysis of immunostaining of CD31, CD45 and Nestin in the whole organoid by two-photon-microscopy. Z-dimension 845 µm. a)-c) Representative images from *n* = 3 independent experiments. **d)** Analysis of LepR expression on CD271+ mesenchymal cells by flow cytometry. **e)** Quantitative analysis of CD271^+^ cells expressing LepR; *n* = 3 independent differentiations. Data are presented as mean value ± SEM. **f)** Z-projection of whole-mount organoid. Arrows indicate enveloped structure enclosing the whole organoid. **g)** Immunohistochemical staining of paraffin embedded organoid section for CD34. **h)** Interconnectivity of vascular-like network computed with Angiotool. f) -k) Representative images from *n* = 3 independent experiments. **i)** TEM image analysis of endothelial cells connected by tight junctions (TJ), Weibel-Palade body (asterisk) and pericyte (P). Representative micrograph of sections from *n* = 2 independent experiments. **j)** Hematoxylin-Eosin (HE) staining of different regions of histological sections with morphologically hematopoietic cells within a lumen of vessel-like structures. Representative images from *n* = 3 independent experiments. **k)** Analysis of organoid sections showing endothelial cells (E) encompassing round cells resembling myeloid cells (M) by TEM. Representative micrograph of sections from *n* = 2 independent experiments. **l)** Orthogonal 2D z-projection of vessel structure of bottom image in Fig. 2b) in xz and yz direction. Note lumen formation indicated by arrows. **m)** Horizontal view of representative z-stack of BMO by confocal microscopy related to Fig. 1h and surface rendering for CD31 and CD45. Still of Supplementary Video 5. **n)** Surface rendering of confocal imaging showing CD31^+^ vessels lined by Nestin expressing cells and enclosing CD45^+^ hematopoietic cells inside the vessel. Still of Supplementary Video 6. Scale bars a), c), f), h) 100µm, b) 50 µm, g), 200µm, i), k) 2 µm, j), l) 20µm, m), n) 10µm. Source data

The BM niche sustains a dynamic interplay between perivascular stromal cells and vascular ECs, orchestrating a microenvironment crucial for hematopoiesis and maintaining HSC functions^2^. Perivascular platelet-derived growth factor beta (PDGFRβ)^+^ mural cells cover ECs, thereby supporting and promoting vasculogenesis^20^. Confocal microscopy of BMOs revealed a vessel-like network of CD31^+^ ECs covered by PDGFRβ^+^ pericytes (Fig. 2a and Extended Data Fig. 3a,b). Interestingly, the formation of PDGFR-β^+^ pericytes changed during the differentiation process. Whereas on day 10 of differentiation, finger-like extensions of PDGFR-β^+^ pericytes were adjacent to ECs (Fig. 2b), upon organoid maturation on day 21 PDGFR-β^+^ pericytes were enwrapping CD31^+^ cells (Fig. 2b,h and Supplementary Fig. 4a), resembling pericytes surrounding endosteal arterioles and Type-H vessels in mice^21^. Next, we examined the spatial distribution of CXCL12^+^ cells, crucial for retention and maintenance of HSCs and hematopoietic progenitors in the BM niche^22^. Remarkably, BMOs contained a perivascular network of CXCL12^+^ cells (Fig. 2c and Extended Data Fig. 3b) with extending protrusions towards the endothelium (Fig. 2d,h and Supplementary Fig. 4a), reminiscent of CXCL12-abundant reticular (CAR) cells^6,22^. Nestin-expressing perivascular mesenchymal stem cells, known to support and regulate hematopoiesis^23^, are located in endosteal regions in spatial association with HSCs and possess multilineage and self-renewal capacity in mice^23^ and human fetal BM^24^. We identified Nestin^+^ stromal cells in spatial relationship to CD31^+^ vessels and CD45^+^ hematopoietic cells in our BMOs by confocal and two-photon microscopy (Fig. 2e,f,h; Extended Data Fig. 3c and Supplementary Video 3). Confocal microscopy revealed Nestin^+^ processes of perivascular cells lining the vascular structures as seen in BM in vivo (Fig. 2e,h and Supplementary Fig. 4a)^23,24^. Leptin receptor (LepR), an additional key marker for mesenchymal niche cells in the BM^25^, was distributed rather broadly on stromal cells (Fig. 2g,h and Supplementary Fig. 4a). In addition, we confirmed surface expression of LepR in around 69% of mesenchymal cells (CD45^−^CD31^−^CD271^+^) (Extended Data Fig. 3d,e), validating the presence of LepR-expressing cells in the BMO.

Next, we aimed at characterizing the vessel structure^21,25,26^. By applying clearing methods before imaging (Supplementary Fig. 4b) and subsequent 3D surface rendering, we confirmed the presence of vascular structures throughout the whole organoid (Fig. 2i, Supplementary Fig. 4c and Supplementary Video 4). ECs formed an envelope around the BMO (Extended Data Fig. 3f) and were also partially positive for CD34, as evidenced by immunohistochemical staining of BMO sections (Extended Data Fig. 3g). Quantitative assessment of vascular structures^27^ confirmed complex network connectivity (mean branching index 186 junctions mm^−2^) (Extended Data Fig. 3h and Supplementary Fig. 4d,e). Importantly, CD31^+^ ECs were covered by a Collagen IV (Col IV)^+^ basement membrane (Fig. 2j). Transmission electron microscopy (TEM) revealed ECs to be connected by numerous tight junctions (Fig. 2k and Extended Data Fig. 3i). Moreover, ECs contained Weibel–Palade bodies and were enclosed by pericytes (Fig. 2l and Extended Data Fig. 3i). Orthogonal two-dimensional analysis documented the formation of a lumen in the vascular network (Extended Data Fig. 3l), which was confirmed by TEM of organoid sections (Fig. 2l). Remarkably, microscopic analysis of hematoxylin-eosin (H/E) stained organoid sections and TEM disclosed capillary-like structures containing round hematopoietic cells in the vessel lumen (Fig. 2m,n and Extended Data Fig. 3j,k). This observation was corroborated by 3D surface rendering of confocal microscopic images demonstrating CD31^+^ vessels enclosing CD45^+^ hematopoietic cells (Fig. 2o; Extended Data Fig. 3m,n and Supplementary Videos 5 and 6). These data support the notion that the vascular network in the BMO consists of bona fide blood vessels encompassing hematopoietic cells in their lumen. Together, these findings indicate that the endothelial and mesenchymal BMO compartments recapitulate key structural features and cell compositions of the human BM niche.

### Functional properties of MSPCs and vascular network

**Extended Data Fig. 4:**
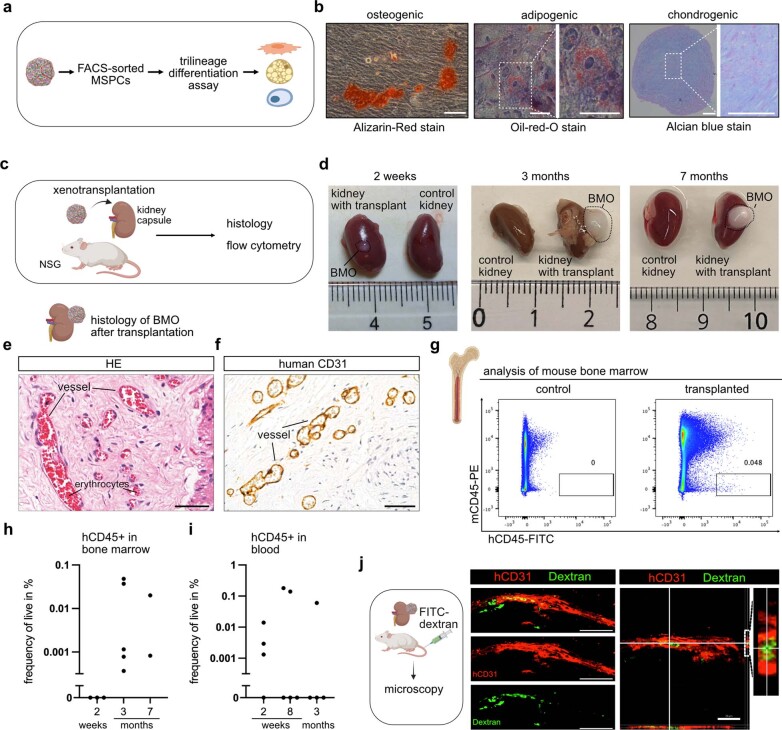
Functional properties of MSPCs and BMO kidney capsule xenotransplantation to test vascular functionality. **a)** Scheme representing the trilineage differentiation assay of FACS-sorted BMO-derived MSPCs. MSPCs were cultured in either osteogenic, adipogenic or chondrogenic differentiation medium for at least 21 days. **b)** Osteogenic, adipogenic and chondrogenic differentiation was visualized by Alizarin Red-S, Oil-red-O or Alcian-Blue staining, respectively; *n* = 4 independent experiments with iPSC line 1 and 2. **c)** Scheme representing xenotransplantation of BMOs under the kidney capsule of NSG mice and follow-up analysis. **d)** Macroscopic morphology of xenotransplants attached to kidney and untransplanted control kidney after 2 weeks, 3 months and 7 months showing further growth *in vivo*. **e)** Hematoxylin-Eosin-stained section of paraffin-embedded organoid 3 months after transplantation, *n* = 3 mice. **f)** Immunohistochemistry of human-specific CD31 antibody to determine BMO origin of vessels at 3 months, *n* = 3 mice. **g)** Representative plot of flow cytometric analysis for presence of human CD45^+^ cells within murine bone marrow in transplanted mice and control mice. Frequency of hCD45+ cells in **h)** bone marrow and **i)** blood of transplanted mice at indicated time points after transplantations, bone marrow: 2 weeks *n* = 3 mice, 3 months *n* = 5 mice, 7 months *n* = 2 mice; blood: 2 weeks *n* = 4 mice, 8 weeks *n* = 5 mice 3 months *n* = 4 mice. **j)** Immunofluorescence analysis of BMO transplants after intravenous injection of FITC-labeled dextran, *n* = 3 mice. Scale bars in b), e), f) 50µm, j) left 50µm, right 20µm. Source data

To test the multipotency of BMO-derived immunophenotypic MSPCs, we performed trilineage differentiation assays (Extended Data Fig. 4a). Remarkably, when placed into the respective conditioned medium, isolated MSPCs from both iPSC1- and iPSC2-derived BMOs had the capacity to differentiate into osteogenic, adipogenic and chondrogenic cells, as visualized by Alizarin Red staining of calcium deposits, Oil-red-O staining of lipid vacuoles and Alcian-blue staining of glycosaminoglycans, respectively (Extended Data Fig. 4b and Supplementary Fig. 5a,b). These findings indicate the existence of MSPCs in the BMO.

To investigate functional properties of the vascular network in vivo, we transplanted mature BMOs under the renal capsule of immunodeficient NOD/SCID/IL2Rγ^null^ (NSG) mice^16,28^ (Extended Data Fig. 4c). Macroscopic analysis showed growth up to 7 months after transplantation (Extended Data Fig. 4d). Flow cytometry analysis at 3 months after transplantation indicated continued presence of the main BMO cell types, albeit at low numbers (Supplementary Fig. 5e,f). Histological analysis of organoid sections after transplantation displayed blood vessels filled with erythrocytes (Extended Data Fig. 4e). We confirmed their BMO origin by human-specific CD31 staining (Extended Data Fig. 4f and Supplementary Fig. 5c,d). Flow cytometry analysis at 3 and 7 months after transplantation detected human CD45^+^ (hCD45^+^) cells in the BM of a proportion of transplanted mice (Extended Data Fig. 4g,h and Supplementary Fig. 5g). Moreover, hCD45^+^ were detected at low frequencies in the blood of transplant recipients (Extended Data Fig. 4i), indicating organoid access to the murine vasculature permissive for circulation of BMO-derived human CD45^+^ cells. To verify vascular interconnection, we intravenously injected fluorescently labeled dextran into NSG mice hosting the transplanted BMOs (Extended Data Fig. 4j). Using confocal microscopy, we detected dextran in the lumen of hCD31 positive vessels, confirming vascular connection (Extended Data Fig. 4j and Supplementary Fig. 5h,i).

### Granulopoiesis

**Fig. 3:**
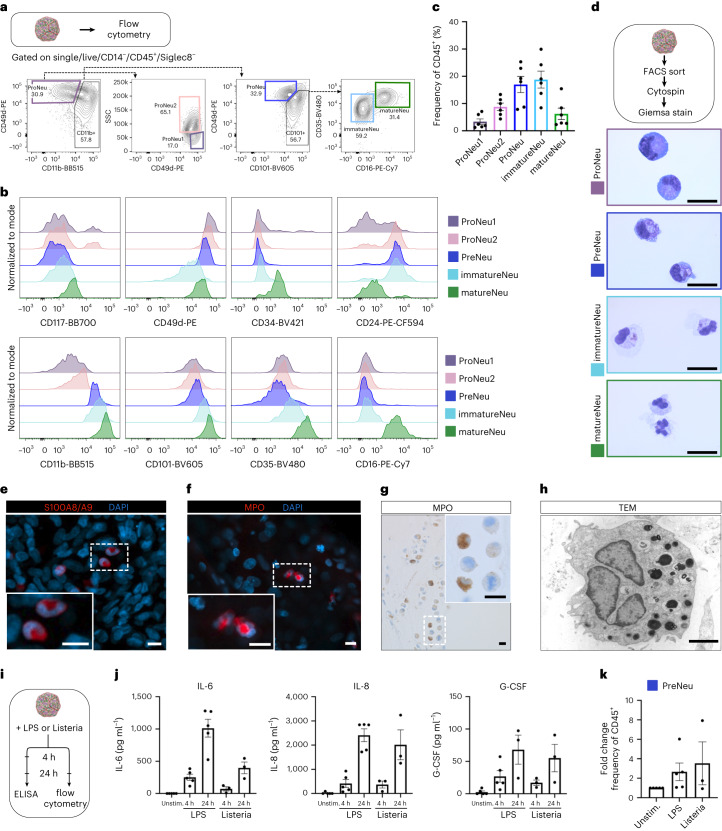
BMOs model granulopoiesis. **a**, Gating scheme for neutrophil differentiation analysis of dissociated BMOs on day 21 of differentiation by flow cytometry. **b**, Histogram of surface marker expression of distinct neutrophil progenitor stages until mature neutrophil-like state of BMO-derived neutrophils. **c**, Quantification of the frequency of neutrophil progenitor subpopulations in the CD45^+^ population; *n* = 6 independent experiments. **d**, May–Gruenwald–Giemsa staining of BMO-derived sorted neutrophil progenitors and mature neutrophil-like cells. Representative images from *n* = 2 independent experiments. **e**,**f**, Confocal imaging of immunostaining of S100A8/A9 (**e**) and MPO (**f**) in cells with banded/segmented nucleus. Insets, magnified images of the rectangle areas. DAPI, 4,6-diamidino-2-phenylindole. **g**, Analysis of MPO expression of a BMO section by immunohistochemistry. Inset, magnified image of the rectangle area. Images in **e–g** are representative images from *n* = 3 independent experiments. **h**, TEM image of typical morphology of a cell resembling a neutrophil granulocyte in the organoid. Representative micrograph of sections from *n* = 2 independent experiments. **i**, Experimental layout used to model inflammation. ELISA, enzyme-linked immunosorbent assay. **j**, Cytokine levels in supernatant of stimulated and unstimulated BMOs, LPS *n* = 5 independent experiments and Listeria (*L. monocytogenes*), *n* = 3 independent experiments. Unstim., unstimulated. **k**, Quantification of the fold change of the PreNeu population upon stimulation in BMOs; LPS, *n* = 5 independent experiments and Listeria *n* = 3 independent experiments. Scale bars, **d**, 20 µm; **e**, **f**, 10 µm; **g**, 50 µm (inset 10 µm); h, 2 µm. Data in **c**, **j** and **k** are presented as mean values ± s.e.m. Source data

Next, we investigated maturation of neutrophil granulocytes by flow cytometry^29–31^ (Fig. 3a,b and Supplementary Fig. 6a,b). Based on expression of specific cell surface markers^29–31^, neutrophil progenitor stages ProNeu1 (CD14^−^CD45^+^Siglec8^−^CD11b^−^CD49d^high^SSC^low^), ProNeu2 (CD14^−^CD45^+^Siglec8^−^CD11b^−^CD49d^high^SSC^high^) and PreNeu (CD14^−^CD45^+^Siglec8^−^CD11b^+^CD49d^+^CD101^−^), as well as immatureNeus (CD14^−^CD45^+^Siglec8^−^CD11b^+^CD49d^low^CD101^+^CD35^+^CD16^−^) and matureNeus (CD45^+^Siglec8^−^CD11b^+^CD49d^low^CD101^+^CD35^+^CD16^+^) were identified in BMOs derived from iPSC1 (Fig. 3a–c and Supplementary Fig. 7a,b) and iPSC2–5 (Supplementary Fig. 7). Light microscopic analysis of flow-sorted and Giemsa-stained cells showed that defined stages of BMO-derived neutrophil granulocytes morphologically resemble their counterparts in the human BM (Fig. 3d and Supplementary Fig. 6c). Immunofluorescent staining for the key neutrophil markers S100A8/A9 and myeloperoxidase (MPO) confirmed expression of both S100A8/A9 and MPO in cells in the BMOs, some of them exhibiting a banded or segmented nucleus indicative of myeloid maturation (Fig. 3e,f). Immunohistochemical studies validated expression of MPO by myeloid cells (Fig. 3g), and TEM analysis revealed the existence of cells with electron-dense cytoplasmic granules, a segmented nucleus and heterochromatin formation at the nuclear margin (Fig. 3h), which are characteristic features of neutrophil granulocytes. These findings indicate that the BMO niche promotes maturation of neutrophil granulocyte-like cells without the addition of lineage-directing cytokines, such as granulocyte colony stimulating factor (G-CSF). To examine the responsiveness of the granulopoietic compartment in the BMOs to inflammatory stimuli, as reported for emergency granulopoiesis in mice^29,32^, we simulated inflammation by addition of lipopolysaccharide (LPS) derived from gram-negative *Escherichia coli* bacteria or heat-killed gram-positive *Listeria monocytogenes* to the BMOs (Fig. 3i). Subsequent analysis of the BMO supernatant revealed increased secretion of the inflammatory cytokines IL-6, IL-8 and G-CSF after 4 h, which increased after 24 h (Fig. 3j). Simultaneously, analysis of the neutrophil progenitor subpopulations showed an increase in the PreNeu subset compared with unstimulated controls after 24 h (Fig. 3k and Supplementary Fig. 6d). These results are consistent with findings in mice^29^ and suggest that BMOs provide a model to study emergency granulopoiesis in a human context^32^.

### Cell type analysis by single-cell transcriptomics

**Fig. 4:**
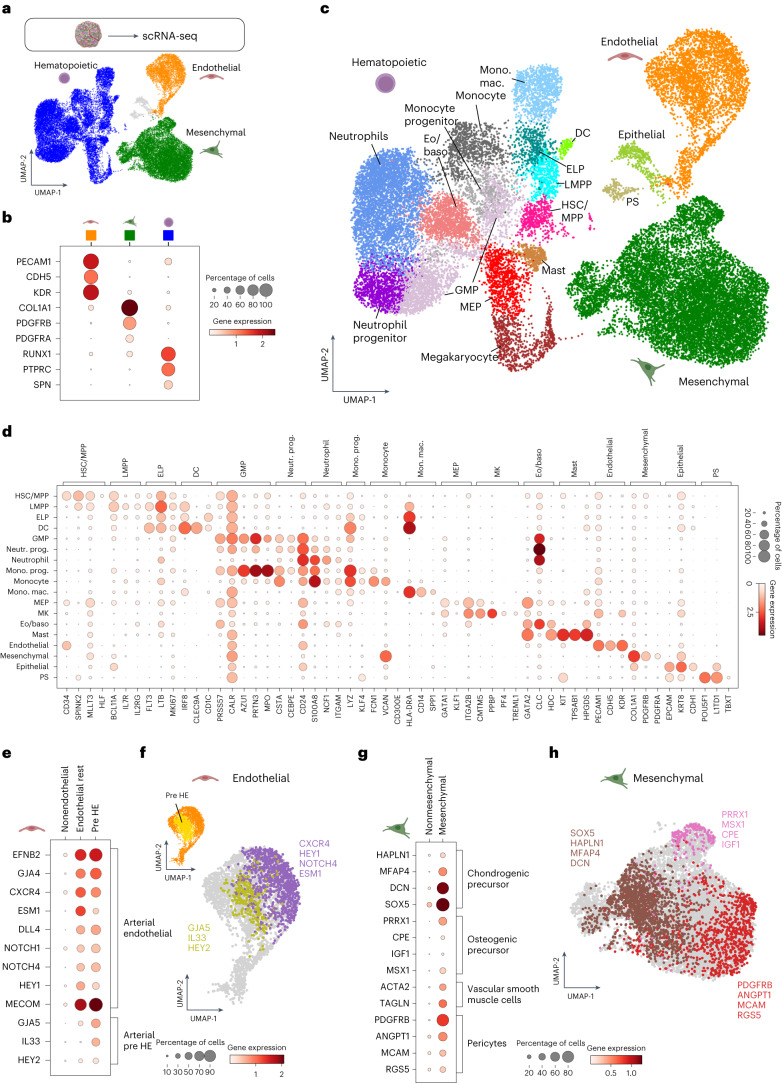
Single-cell transcriptomic analysis of BMOs identifies diverse cell populations. **a**, Coarse-grained clustering of scRNA-seq data reveals three main populations comprising endothelial, hematopoietic and mesenchymal cells. **b**, Expression of characteristic markers indicative of the three main populations. **c**, Uniform manifold approximation and projection (UMAP) projection of a total number of 31,040 cells colored according to detailed cell type annotations. eo/baso, eosinophil/basophil; GMP, granulocyte/monocyte-progenitor; MPP, multipotent progenitor; LMPP, lymphoid-primed multipotent progenitors; MEP, megakaryocyte/erythroid progenitor, MK, megakaryocyte; mono. mac., monocytoid macrophage; PS, primitive streak-like. **d**, Expression of marker genes for annotation of hematopoietic lineages. Neutr. prog., neutrophil progenitor; mono. prog., monocyte progenitor. **e**, Expression of marker genes for endothelial subtypes. **f**, UMAP projection for arterial endothelial and pre-HE cells coexpressing displayed marker genes. **g**, Expression of marker genes for mesenchymal cell clusters. **h**, UMAP projection of distinct mesenchymal subsets coexpressing marker genes.

To characterize the cellular composition of the organoid model on a molecular level, we performed single-cell RNA sequencing (scRNA-seq) of dissociated BMOs from two independent batches of differentiation (Supplementary Fig. 8a,b). After quality control, our dataset comprised a total number of 31,040 cells (Fig. 4a,c). Unsupervised clustering revealed three main distinct populations, which were identified as hematopoietic, endothelial and mesenchymal lineages, respectively (Fig. 4a,b). When compared with an atlas of human gastrulation^33^, a minor cluster of cells was enriched for primitive streak genes (Fig. 4c,d), suggesting the presence of a small subset of immature progenitor cells in the organoid. Additionally, we noted the presence of a small epithelial cell cluster (Fig. 4c,d). We observed a high degree of consistency in the overall cell type composition in comparison with the clustering of immunophenotypic data (Fig. 1d and Fig. 4a), with minor shifts in the frequency distribution of the individual cell types (Supplementary Fig. 8c and Fig. 1e).

**Extended Data Fig. 5:**
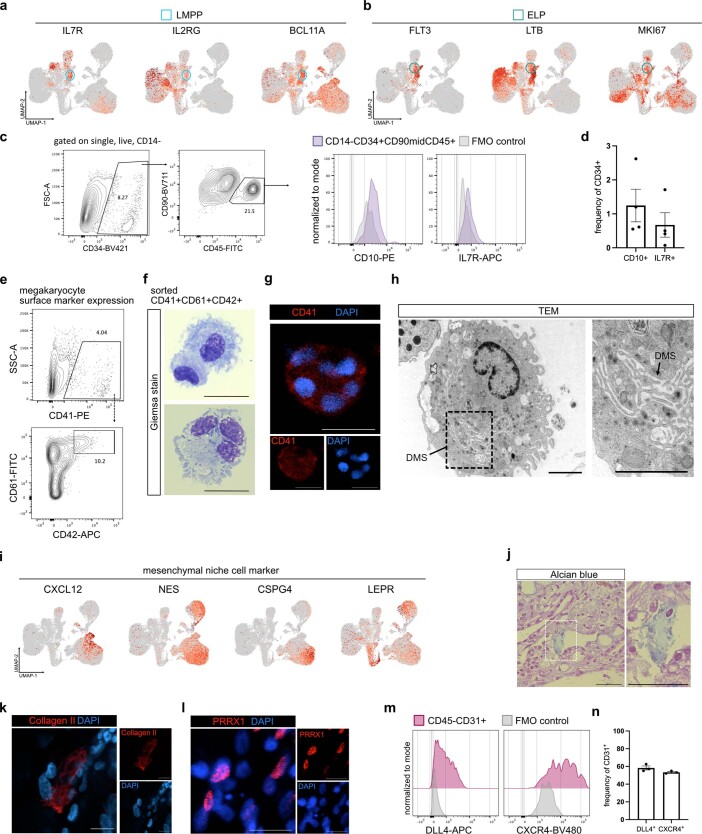
Identification of lymphoid progenitor cells and megakaryocyte-like cells and analysis of mesenchymal and endothelial cell population. **a)** UMAP projection of LMPP marker genes; LMPP, lymphoid-primed multipotent progenitors. **b)** UMAP projection of ELP marker genes; ELP, early lymphoid progenitor. **c)** CD10 and IL7R expression on a CD34^+^CD90^mid^CD45^+^ subset by flow cytometry. **d)** Quantitative analysis of CD10 and IL7R expression on a CD34^+^CD90^mid^CD45^+^ subset for *n* = 4 independent experiments. Data are shown as mean ± SEM. **e)** Detection of CD41^+^CD61^+^CD42^+^ cells by flow cytometry of dissociated BMOs. **f)** Analysis of Giemsa-stained cytospin preparations of sorted CD41^+^CD61^+^CD42^+^ cells, *n* = 2 independent experiments. **g)** Immunofluorescence analysis of multinucleated CD41+ cell within the BMO, *n* = 3 independent experiments. **h)** TEM imaging analysis of organoid section displaying a large cell with a characteristic demarcation membrane system (DMS). Representative micrograph of sections from *n* = 2 independent experiments. **i)** UMAP projection of mesenchymal niche marker gene expression. **j)** Alcian blue positive spots in sections of BMOs at day 45 of differentiation. **k)** Rare Collagen II expressing cells in the BMO at day 21 of differentiation. **l)** Analysis of nuclear expression of PRRX1 in mesenchymal cells by confocal microscopy. **j)-l)** *n* = 3 independent experiments. **m)** Representative histogram for expression of arterial surface markers DLL4 and CXCR4 on CD31^+^ endothelial cells and **n)** quantitative frequencies of DLL4 and CXCR4 on CD31^+^ endothelial cells for *n* = 3 independent experiments. Data are presented as mean values ± s.e.m. Scale bars in f), g) 20µm, h) 2 µm, j), k), l) 20µm. Source data

Using previously published molecular signatures of human fetal BM^34^ and other published datasets^35–37^, we identified principal hematopoietic cell types in the hematopoietic cluster, marked by expression of *PTPRC* (CD45), *SPN* (CD43) and *RUNX1*. This cluster comprised not only myeloid progenitors, as well as neutrophils, monocytes and macrophages, but also eosinophil/basophil, mast cells, dendritic cells (DC), megakaryocytic and, importantly, lymphoid progenitor cells (Fig. 4b,d). These latter express markers of lymphoid-primed multipotent progenitors, for example, *IL7R* and *BCL11A*^34^, as well as marker genes that are indicative of early lymphoid progenitors such as *LTB* and *FLT3* (ref. ^34^) (Fig. 4d and Extended Data Fig. 5a,b). We validated the expression of IL-7 receptor (IL7R) and CD10 corresponding to markers for lymphoid progenitors^38,39^ on a subset of CD34^+^CD90^mid^CD45^+^ cells by flow cytometry (Extended Data Fig. 5c,d). Moreover, we confirmed the presence of CD41^+^CD61^+^CD42^+^ megakaryocyte-like cells by a series of various methods revealing immunophenotypic and morphological characteristics of megakaryocytes (Extended Data Fig. 5e–h).

Cells in the endothelial cluster, defined by expression of *PECAM1* (CD31), *CDH5* (VE-Cadherin) and *KDR* (VEGFR-2), also expressed canonical genes of arterial ECs, such as *EFNB2*, *GJA4* and *CXCR4* (ref. ^37^). Notably, these ECs also highly expressed Notch signaling pathway genes (*DLL4*, *NOTCH1*, *NOTCH4*, *HEY1*), which had previously been reported to be important for definitive hematopoiesis in vivo^37^ and differentiation of definitive HE from PSCs in vitro^40^. Accordingly, a subpopulation was characterized by marker genes of arterial pre-HE^37^ (Fig. 4e,f). We confirmed expression of arterial surface markers (DLL4, CXCR4) on CD31^+^ ECs by flow cytometry (Extended Data Fig. 5m,n).

In the mesenchymal cluster, defined by *COL1A1* (Collagen I), *PDGFRA* and *PDGFRB*, we could distinguish pericytes (*PDGFRB*, *ANGPT1*, *MCAM*, *RGS5*)^20^ and vascular smooth muscle cells (*ACTA2*, *TAGLN*)^6,36^ (Fig. 4g,h). Moreover, in line with our immunofluorescence results, cells in the mesenchymal cluster expressed known HSC-supporting genes, such as *CXCL12* (ref. ^22^), *NES* (Nestin)^23^, *CSPG4* (NG2)^26^ and *LEPR*^25^ (Extended Data Fig. 5i). Furthermore, we identified chondrogenic precursor cells expressing *HAPLN1* (ref. ^34^), *SOX5* (ref. ^41^) and *DCN*^42^, as well as osteogenic precursors marked by the transcription factors *PRRX1* (ref. ^43^), *MSX1* (ref. ^44^) and *CPE*^34^ (Fig. 4g,h). Histological staining of organoid sections from day 45 of differentiation revealed the presence of rare Alcian-blue positive cells with an oval shaped nucleus, indicative of mucopolysaccharides (Extended Data Fig. 5j). Additionally, we detected expression of Collagen II, which is associated with chondrocytes, by immunofluorescence microscopy. (Extended Data Fig. 5k). The nuclear expression of PRRX1 in mesenchymal cells was validated through immunofluorescence microscopy (Extended Data Fig. 5l).

Overall, the single-cell transcriptomic data supports the notion that BMOs contain highly diverse cell populations reminiscent of fetal BM at the molecular level.

### Characterization of HSPCs in BMOs

**Fig. 5:**
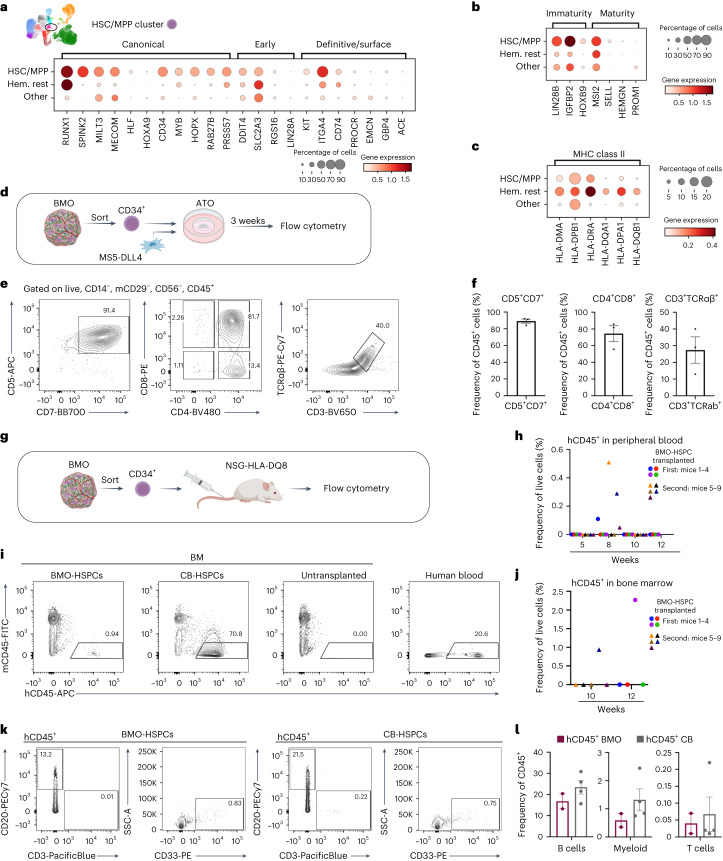
Molecular signature resembling fetal HSCs, lymphoid potential and engraftment potential of BMO-derived HSPCs. **a**–**c**, scRNA-seq of BMOs, revealing expression of canonical, early and definitive/surface HSC marker genes (**a**), marker genes for immature and mature states of HSCs (**b**) and HLA-class II molecule genes indicative of maturation (**c**). Hem., hematopoietic. **d**, Experimental layout for generation of ATOs with BMO-derived CD34^+^ cells and their analysis by flow cytometry. **e**, Flow cytometric analysis of surface marker expression of live, CD14^−^, mCD29^−^, CD56^+^ and CD45^+^ cells. **f**, Quantification of frequencies of lymphoid populations shown in **e**, *n* = 3 independent batches of BMO and ATO differentiation. **g**, Experimental layout for testing of in vivo engraftment potential. **h**, Quantification of frequencies of human chimerism in peripheral blood of transplanted mice at weeks 5, 8, 10 and 12. **i**, Flow cytometric analysis of human chimerism (hCD45^+^ cells) in BM of mice transplanted with BMO-derived HSPCs, CB controls or nontransplanted controls. **j**, Quantification of frequencies of hCD45^+^ cells in BM of mice transplanted with BMO-derived HSPCs after 10 and 12 weeks; *n* = 2 independent transplantations with two independent batches of BMO differentiation. **k**, Flow cytometric analysis of surface marker expression of hCD45^+^ engrafted cells in BMO-derived HSPC transplant and CB controls. **l**, Quantification of frequencies of hCD20^+^ (B cells), hCD33^+^ (myeloid cells) and hCD3^+^ (T cells) of engrafted hCD45^+^ cells in BM of *n* = 2 BMO-derived HSPC-transplanted and *n* = 4 CB-transplanted animals. Data in **f** and **l** are presented as mean values ± s.e.m. Source data

In our scRNA-seq dataset we also identified a distinct population of cells expressing signature genes of human fetal HSCs/MPPs^34,37,45,46^ (Fig. 4c,d). During human embryonic development, definitive long-term HSCs first emerge in the aorta-gonad-mesonephros (AGM) region, migrate to the liver and ultimately colonize the fetal BM at around 11–12 weeks post conception^3,34,47^. We compared gene expression signatures in BMOs with previously reported molecular signatures of human fetal HSCs in yolk sac, AGM, fetal liver and fetal BM^34,37,45,46^. Our findings revealed that BMO-derived HSPCs expressed key transcription factors associated with definitive HSCs, which are essential for HSC self-renewal (*MLLT3*), HSC maintenance (*MECOM*) and undifferentiated HSPCs (*HLF*) (Fig. 5a). *HLF* emerged as the most distinctive marker for this cluster in our dataset, in line with human fetal HSCs in the AGM^37^. Additionally, canonical HSC marker genes (*RUNX1*, *SPINK2*, *HOPX*, *RAB27B*, *MYB*), as well as surface markers indicative of definitive HSCs (*KIT*, *CD74, ITGA4*) exhibited high expression levels (Fig. 5a). While levels of medial HOX genes (*HOXA9*, *HOXA10*) were low, posterior HOX genes (*HOXB9*, *HOXB7*) were prominently expressed. BMO-derived HSPCs expressed not only embryonic genes (*LIN28B*, *IGFBP2*), but also maturity markers, which are present only from the fetal liver stage onwards and associated with maturation of HSCs^37^ (Fig. 5b). The latter include the transcription factor *MSI2* and surface marker *SELL* (CD62L) (Fig. 5b), as well as expression of major histocompatibility complex class II molecules (Fig. 5c). Together our data support the notion that the multilineage hematopoietic compartment in BMOs, including lymphoid progenitor cells, and the presence of arterial-type ECs recapitulates definitive hematopoiesis^47,48^.

To examine the multilineage differentiation capacity of BMO-derived HSPCs, we performed colony-forming unit (CFU) assays (Supplementary Fig. 9a). FACS-sorted HSPCs gave rise mainly to granulocyte-macrophage progenitor cells (CFU-GM), but also multipotential granulocyte, erythroid, macrophage and megakaryocyte progenitor cells (CFU-GEMM) and erythroid progenitors (BFU-E) (Supplementary Fig. 9a,b). Isolated HSPCs from iPSC2-derived BMOs also gave rise to CFU-GEMM and CFU-GM, yet not to typical BFU-E colonies (Supplementary Fig. 9c,d). Second, to examine the lymphoid differentiation potential of BMO-derived HSPCs^48^, we adapted an artificial thymic organoid (ATO) differentiation system, which has been established previously for primary human HSPCs^49^ and PSCs^50^ (Fig. 5d and Supplementary Fig. 9e). Following 3 weeks of ATO culture, we observed the emergence of CD5^+^CD7^+^ lymphoid cells. Notably, most of these cells (around 74%) were CD4^+^CD8^+^ double-positive and around 27% expressed CD3 and TCRαβ, phenotypically resembling T cells (Fig. 5e,f and Supplementary Fig. 9f). Finally, to determine engraftment potential of BMO-derived HSPCs in vivo, we transplanted sorted BMO-derived CD34^+^ cells or human cord blood (CB) CD34^+^ cells as controls via intravenous injection into immunodeficient major histocompatibility complex-II-deficient NSG-HLA-DQ8 transgenic mice (NSG-HLA-DQ8)^51^ (Fig. 5g). Serial blood draws were performed at 5, 8 and 10 weeks post transplantation, followed by an analysis of human chimerism by flow cytometry. Eight weeks after transplantation, human CD45^+^ cells were detectable in the peripheral blood in 44% of the transplanted animals, indicating partial engraftment (Fig. 5h). Flow cytometry analyses of the BM revealed the presence of hCD45^+^ cells in one out of five mice after 10 weeks (0.94% hCD45^+^) and one out of four mice after 12 weeks (2.27% hCD45^+^) (Fig. 5i,j and Supplementary Fig. 9g,h). Human CD45^+^ cells in recipient mice contained CD20^+^ cells (B cell marker), as well as CD33^+^ cells (myeloid cell marker). Similar frequencies were obtained in mice that were transplanted with CB CD34^+^ cells. We also detected a few hCD45^+^ cells expressing CD3, indicative of T cells (Fig. 5k,l and Supplementary Fig. 9i). These data provide evidence for engraftment and multilineage differentiation potential of the HSPCs generated in our BMO model.

### Modeling hematopoietic development and genetic disease

**Fig. 6:**
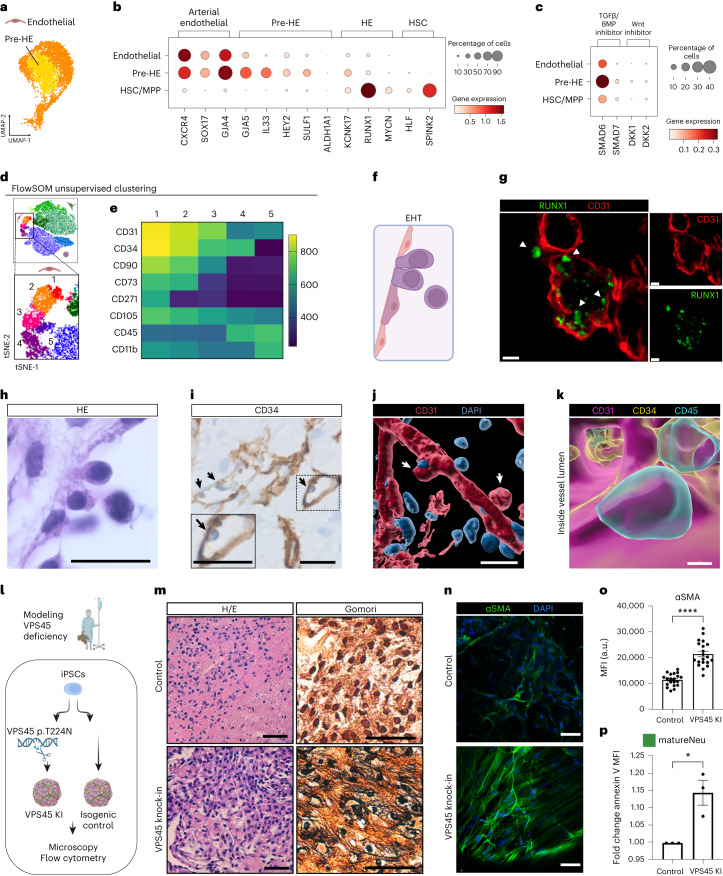
Modeling features of hematopoietic development and genetic disease in BMOs. **a**, UMAP projection of pre-HE subpopulation in the endothelial cluster. **b**, Dotplot shows expression of key marker genes for arterial ECs, pre-HE, HE and HSPCs during EHT. **c**, Dotplot for expression of TGFβ/BMP or Wnt inhibitor genes in different subsets. **d**, Unsupervised clustering of flow cytometry populations using the FlowSOM algorithm reveals heterogeneity in the endothelial cluster. **e**, Heatmap of mean fluorescence intensity (MFI) of indicated surface marker expression in BMO cell clusters 1–5 identified by FlowSOM algorithm**. f**, Scheme depicting morphological changes during transition from HE to HSPCs. **g**, Coexpression of RUNX1 and CD31 in clusters of cells by confocal imaging. RUNX1^+^ cells indicated by round morphology (arrow) are attached to endothelial layer. **h**,**i**, HE- (**h**) andCD34- (**i**) stained organoid section indicating budding of hematopoietic cells from the endothelial wall. **j**, Surface rendering of immunostaining of CD31 in the BMO at day 10. **k**, Surface rendering of immunostaining of CD31, CD34 and CD45 inside a vessel lumen in the BMO at day 10; still frame from Supplementary Video 7. Images in **g**–**k** are representative images from *n* = 3 independent experiments. **l**, Modeling of VPS45 deficiency in BMOs and follow-up analysis. Schematic overview of gene editing and experimental set-up to create an isogenic VPS45 mutant iPS cell line. **m**, Histological comparison of control and VPS45 mutant BMOs by H/E and Gomori stain reveals reticulin fibrosis in VPS45 mutant BMOs; *n* = 8 organoids for each condition of two batches. **n**, Alpha SMA expression in control and VPS45^−^ mutant BMOs analyzed by immunofluorescence. **o**, Quantification of MFI of SMA expression; four different regions of *n* = 5 organoids per condition of two batches. ****P < 0.0001, unpaired two-tailed *t*-test. **p**, Quantification of Annexin MFI on matureNeus by flow cytometry; *n* = 3 independent experiments. *P = 0.0155, unpaired two-tailed *t*-test. Data in **o** and **p** are presented as mean values ± s.e.m. Scale bars, **g**, **j**, 20 µm; **h**, **i**, **m**, **n**, 50 µm; **k**, 8 µm. Source data

Having identified arterial pre-HE^37^ resembling cells by scRNA-seq (Fig. 4e,f and Fig. 6a), we next set out to study endothelial-to-hematopoietic transition (EHT). During human embryonic development, definitive HSCs emerge from specialized ECs lining the ventral wall of the dorsal aorta^37,47,52^. This involves the transition of arterial ECs to pre-HE and HE towards HSCs, characterized by the sequential downregulation of arterial endothelial genes and the upregulation of HSC-specific genes^37^. We identified a subset of cells in the endothelial cluster expressing markers associated with pre-HE^37^, including *IL33*, *SULF1*, *GJA5* and *HEY2*, although *ALDH1A1* expression was limited (Fig. 6b). Whereas we could not distinguish a specific HE subcluster, cells in the pre-HE cluster exhibited key HE genes such as *KCNK17* (ref. ^37^) and displayed low levels of *RUNX1*, indicating initiation of hemogenic transition (Fig. 6b). Moreover, while Wnt inhibitor genes (*DKK1*, *DKK2*) were low, TGFβ/BMP-inhibitor genes (*SMAD6*, *SMAD7*) previously reported to be essential for EHT^37^, were upregulated in the pre-HE cluster (Fig. 6c). Thus, we identified molecular signatures of EHT processes in our BMO. To capture intermediate cellular differentiation states in our flow cytometry data, we analyzed protein expression by unsupervised clustering using the FlowSOM algorithm^53^ (Fig. 6d,e). The endothelial compartment contained additional subpopulations. Cluster 3 cells coexpressed CD31, CD34, CD90 (Thy1)^54^ and CD105 (Endoglin)^55^ and were negative for CD45, resembling human HE and HSCs. The adjacent cluster 4, defined by CD31^low^CD34^+^ and CD45^+^ cells, constituted the manually gated HSPC cluster (Fig. 6d,e). Decreasing CD31 expression while upregulating CD34 and CD45 reflects the immunophenotypic sequence of cells that undergo EHT^56^. Morphologically, EHT is characterized by changes of arterial ECs^52^ (Fig. 6f) and expression of the transcription factor RUNX1 (ref. ^57^). Confocal imaging showed distinct clusters of RUNX1-expressing cells with some of these cells coexpressing CD31 and remaining attached to the endothelial wall (Fig. 6g). Furthermore, H/E staining of organoid sections revealed rounding up of cells from the endothelial lining (Fig. 6h). This finding was also observed in histological sections of BMOs stained for CD34 (Fig. 6i). Immunofluorescence of immature organoids (day 10) followed by 3D surface rendering demonstrated CD31^+^ cells budding off from endothelial structures (Fig. 6j) and revealed CD34 expression of vessels, as well as round CD34^+^ cells attached to the endothelial lining inside the vessel lumen (Fig. 6k, Supplementary Fig. 10a and Supplementary Video 7), thus reflecting findings of EHT in vivo^52^. These data suggest that BMOs give rise to arterial-type ECs, pre-HE and HE, which transition into human HSPC-like cells by EHT, thus modeling features of developmental processes in human hematopoiesis.

Finally, we tested whether our BMOs can be used as a model system to recapitulate the phenotype of monogenic BM diseases. Children with vacuolar protein sorting 45 homolog (*VPS45*) deficiency clinically present with neutropenia and myelofibrosis in the first year of life and progress to overt BM failure^58,59^. Their BM is hypercellular and shows myeloid hyperplasia associated with increased apoptosis and functional deficiencies of neutrophils^58,59^. We generated BMOs from an iPSC line, in which the homozygous Thr224Asn mutation in *VPS45* had been introduced by CRISPR–Cas9-mediated gene editing^12^ (Fig. 6l and Supplementary Fig. 10b,c). The composition of hematopoietic as well as niche cells was comparable between isogenic control BMOs and VPS45-mutant BMOs (Supplementary Fig. 10d). In contrast to controls, VPS45-mutant BMOs showed increased deposition of reticulin fibers (Fig. 6m), reminiscent of myelofibrosis in BM biopsies of VPS45-deficient patients^58,59^. This was accompanied by expansion of alpha smooth muscle actin (αSMA) expressing myofibroblast-like stromal cells (Fig. 6n,o), previously described as critical drivers of myelofibrosis^20^. Flow cytometric analysis of VPS45-mutant BMOs showed higher numbers of matureNeus in comparison with controls (Supplementary Fig. 10e) resembling myeloid hyperplasia and a significant increase in AnnexinV expression on VPS45-mutant matureNeus (Fig. 6p and Supplementary Fig. 10f), indicating enhanced apoptosis of this subpopulation—a feature also observed in patients^59^. Thus, BMOs may provide appropriate model systems for dissecting genes and pathways in previously intractable pathomechanisms of BM failure diseases.

## Discussion

We here report the development of complex iPSC-derived human BMOs. Previously, approaches of generating a BMO^60^ or BM-on-a-chip^61^ from primary human cells relied on tedious protocols for isolation of BM cells, which are often not easily obtainable. The BMO protocol offers advantages in simplicity and reproducibility by using off-the-shelf products and commercial iPS cell lines, being serum- and feeder-free and without requirement for hypoxic cultivation conditions. The successful generation of BMOs from renal epithelial cells may bear clinical significance, especially for pediatric patients, where urine samples can serve as a noninvasive, pain-free and efficient source for iPSCs^62,63^.

Nevertheless, BMOs share common limitations associated with iPSC-derived tissues in vitro^64^. Due to cellular immaturity, the BMOs are more similar to fetal rather than adult BM. Furthermore, they lack osseous trabeculae and neuronal cells.

Recently, human PSC-derived hematopoietic organoids^9,65^ generated hematopoietic cells with lymphoid potential, a vascular network and a subset of stromal cells; however, their stromal architecture was not described in detail. Here, advanced imaging techniques revealed a complex architecture of perivascular and endothelial niche cells in the BMOs and functional studies confirmed multipotency of MSPCs and connection of vessel structures in vivo. Additionally, BMOs contained immunophenotypically defined neutrophil progenitor subsets, supported the maturation of granulopoietic cells without addition of cytokines such as G-CSF and exhibited responsiveness to inflammatory stimuli.

Our data suggest that BMOs recapitulate aspects of definitive hematopoiesis, supported by molecular signatures of arterial ECs and pre-HE and lymphoid differentiation capacity of BMO-derived HSPCs. In parallel to our studies, Khan et al. developed human iPSC-derived BMOs, supporting leukemic cell engraftment and disease modeling^10^. Unlike our approach, their strategy yielded predominantly erythromyeloid cell types in the hematopoietic compartment, resembling extraembryonic-type hematopoiesis^47,66^. As precise mesodermal patterning for induction of intraembryonic-type definitive hematopoiesis requires activation of Wnt-signaling and inhibition of activin/nodal-signaling^9,48,65,66^, inclusion of a Wnt-activator (CHIR99021) and activin/nodal-inhibitor (SB431542) in our protocol might be key for differentiation of arterial-type ECs and emergence of HSPCs giving rise to a multilineage hematopoietic compartment in the organoid.

scRNA-seq identified a cell population in BMOs expressing genes of human fetal HSCs, including key HSC-associated factors like *RUNX1*, *SPINK2*, *MLLT3*, *MECOM* and *HLF*. The lack of *HOXA* patterning gene expression in our HSPC cluster, essential for true HSC generation^67^, may result from lack of retinoid acid signaling, reflected by low *ALDH1A1* levels in the pre-HE cluster. However, they displayed canonical HSC genes and, to some extent, expression of fetal liver-associated genes, resembling in part both AGM and fetal liver HSCs^37^. Remarkably, BMO-derived CD34^+^ cells showed engraftment potential upon transplantation into immunodeficient mice, albeit only in a subset of recipients. This is a noteworthy outcome, considering that successful transient engraftment of ex vivo differentiated iPSC-derived HSPCs without transgenic expression of transcription factors has been challenging. Luff et al.^68^ recently reported engraftment of iPSC-derived hemogenic endothelium in murine BM up to 10 weeks. The presence of human chimerism at 10 and 12 weeks posttransplantation in our experiments underscores the encouraging engraftment potential of BMO-derived HSPCs. Addition of retinoic acid to the culture conditions as previously reported^67,68^ might induce a molecularly more mature HSPC population, which could enhance engraftment potential in the future.

Notably, gene-edited iPSC-derived BMOs modeled aspects of an inherited BM failure syndrome by recapitulating features of myelofibrosis alongside a hematopoietic phenotype. Existing models often rely on the addition of fibrosis-inducing agents^10^ or humanized PDX models^69^. Thus, BMOs provide a platform to study genetic diseases in the context of a complex human niche.

Finally, since the lack of immune cells, vasculature and pericytes presents a common limitation of organoid models^70^, another possible application of our method might be the combination of BMOs with other iPSC-derived organoids to form assembloids^70^.

## Methods

### Cell culture

Human iPS cells were cultivated under feeder-free conditions on growth factor-reduced Matrigel (Corning) or Geltrex (Gibco) coated plates in mTeSRplus medium (STEMCELL Technologies). Cells were passaged every 4–5 days using ReLeSR (STEMCELL Technologies). Cells were tested routinely for mycoplasma contamination.

### Differentiation of BMOs

On day −3, embryoid bodies were generated by dissociation of iPSCs into single cells with Accutase (Gibco) for 5 min at 37 °C. The dissociation reaction was stopped with mTeSRplus and cells were collected at 300*g* for 5 min at room temperature (RT). The cells were then resuspended in aggregation medium^17^ (KnockOut DMEM/F12 supplemented with 20% KnockOut Serum Replacement (Gibco), 1% l-glutamine, 1% nonessential amino acids (Gibco), 1% penicillin-streptomycin and 100 µM β-mercaptoethanol) and counted with a hemocytometer. Next, depending on the iPSC line 1.5–4 × 10^6^ cells were resuspended in aggregation medium supplemented with 50 μM Y-27632 (ROCK-Inhibitor, STEMCELL Technologies) and seeded into a low-attachment petri dish. After 24 h, the medium was changed to fresh aggregation medium without ROCK-inhibitor. For the medium change, embryoid bodies were collected in 15 ml canonical tubes by gravitation (15–20 min). Mesoderm was induced on day 0 with mTeSRplus supplemented with 80 ng ml^−1^ BMP4, 4 μM CHIR99021 (Merck Millipore) and 80 ng ml^−1^ VEGF (STEMCELL Technologies) and the plate was placed on a rocking shaker. Generally, embryoid bodies were resuspended once per day until embedding to avoid excess fusion and placed on a rocking shaker. On day 2, medium was replaced by Essential 6 medium (Gibco) supplemented with 80 ng ml^−1^ VEGF, 25 ng ml^−1^ bFGF, 50 ng ml^−1^ SCF and 2 μM SB431542 (Selleckchem). On day 4, embryoid bodies were collected by gravitation (10–15 min) and 60–100 embryoid bodies were embedded into 1 well of a 12-well plate with 1 ml per well of a 4:1 Collagen I (Ibidi) solution—Matrigel mixture. The Collagen I solution was adapted from Wimmer et al.^16^ and recommendations by the manufacturers’ protocol (25 μl 10× DMEM, 68.75 μl ddH_2_O, 6.25 μl 7.5% sodium bicarbonate, 117.8 μl Hams-F12 (Gibco), 4.8 μl HEPES (Gibco), 2.4 μl Glutamax, 150 μl of 5 mg ml^−1^ Collagen type I) and pH strips were used to assure pH 7.4 (1 N NaOH could otherwise be added dropwise to bring the solution to a pH of 7.4, which was almost never needed). For embedding, first a layer of 500 μl per well of Collagen I Matrigel mixture was prepared (375 µl Collagen I solution and 125 µl Matrigel) and polymerized at 37 °C for 30 min to prevent embryoid bodies sinking to the bottom of the dish. Then, the embryoid bodies (in 10–15 µl medium) were resuspended in 500 μl per well of Collagen I Matrigel mixture and a second layer was prepared and polymerized at 37 °C for 2 h. Finally, the embedded embryoid bodies were overlaid with prewarmed Stempro-34 SFM medium (Gibco) supplemented with 80 ng ml^−1^ VEGF, 25 ng ml^−1^ bFGF, 50 ng ml^−1^ SCF and 2 μM SB431542. On day 6, cytokines were switched to 50 ng ml^−1^ VEGF, 50 ng ml^−1^ SCF, 50 ng ml^−1^ IL-3, 50 ng ml^−1^ Flt-3L and 5 ng ml^−1^ TPO. On day 8, cytokines were changed to 25 ng ml^−1^ VEGF, 50 ng ml^−1^ SCF, 50 ng ml^−1^ IL-3, 50 ng ml^−1^ Flt-3L and 5 ng ml^−1^ TPO. On day 10, sprouted embryoid bodies were extracted from the 3D matrix with sterile dissecting tools under the laminar flow hood on an inverse light microscope. Single sprouted EBs were then transferred and further cultured in a U-bottom 96-well-low-attachment plate (PHC Europe). Thereafter, spherical organoids self-assembled and were cultured in Stempro-34 medium supplemented with 25 ng ml^−1^ VEGF, 50 ng ml^−1^ SCF, 50 ng ml^−1^ IL-3, 50 ng ml^−1^ Flt-3L and 5 ng ml^−1^ TPO and half of the medium was refreshed every 3–4 days. All cytokines were purchased from PeproTech unless indicated otherwise.

### Cell lines

The human fibroblast-derived iPS cell line HMGU1 (iPSC1) was provided by the iPSC Core Facility, Institute of Stem Cell Research, Helmholtz Center Munich (hPSCreg IFSi001-A). The VPS45 mutant (Thr224Asn) derivative iPS cell line has been described previously^12^. In brief, two gRNA oligos (T1, 5′-CACCGTTTGAATTCCGTCGGACAG-3′; T2, 5′-AAACCTGTCCGACGGAATTCAAAC-3′) were cloned into the pSpCas9(BB)-2A-GFP (PX458, catalog no. 48138, Addgene) plasmid. The plasmids and the single-stranded oligodeoxynucleotide were cotransfected into HMGU1 iPS cells using the Amaxa Nucleofector II Device (program B-016, Lonza) and the Human Stem Cell Nucleofector Kit 2 (Lonza). GFP-positive cells were sorted and single colonies picked for sequencing and further expansion after confirmation of successful knock-in. The human iPS cell lines 29B5 (iPSC2) and 12C2 (iPSC5) were generated from urine samples using the CytoTune-iPS v.2.0 Sendai Reprogramming Kit (Thermo Fisher) as described previously^63^. Human urine samples from healthy volunteers (29B5, male; 12C2, female) were obtained with written informed consent and processed anonymously. The experimental procedure was ethically approved by the responsible committee on human experimentation (20-122, Ethikkommission Ludwig-Maximilians-University (LMU) Munich). The human female PBMC-derived iPS cell line SCTi003-A (iPSC3) was purchased from STEMCELL Technologies (catalog no. 200-0511). The human male fibroblast-derived iPS cell line WTC-11 (iPSC4) was purchased via the Coriell Institute (catalog no. GM 25256) from the NIGMS Human Genetic Cell Repository. Authentication and assessment of genomic integrity of cell lines were performed by the respective providers.

### Mouse strains

All mice were bred, maintained, examined and euthanized in accordance with institutional animal care guidelines and ethical animal license protocols approved by the regulatory authorities. NOD.Cg-*Prkdc*^*scid*^ *Il2rg*^*tm1Wjl*^/SzJ (NSG) mice were purchased from Charles River Laboratories. NSG mice were housed at the Institute of Molecular Biotechnology (IMBA, Vienna, Austria) under specific pathogen-free conditions in a 12 h light/dark cycle, at 20–22 °C and 45–60% humidity, with food and water ad libitum. NOD.Cg-*Prkdc*^*scid*^ *H2-Ab1*^*b-tm1Doi*^ *Il2rg*^*tm1Wjl*^ Tg(HLA-DQA1,HLA-DQB1)1Dv/SzJ (NSG-HLA-DQ8) mice were a gift from L. Shultz (The Jackson Laboratory). NSG-HLA-DQ8 mice were bred and housed at Zentrale Versuchstierhaltung (ZVH, LMU Klinikum) under specific pathogen-free conditions in a 12 h light/dark cycle, at 20–22°C and 45–60% humidity, with food and water ad libitum. All mouse experiments were performed according to guidelines of the Federation of European Laboratory Animal Science Association (FELASA) and the protocols were approved by the Austrian Federal Ministry of Education, Science and Research (NSG mice) and the Government of Upper Bavaria (Regierung von Oberbayern) (NSG-HLA DQ8 mice).

### Mouse kidney capsule transplantation

BMOs were transplanted under the kidney capsule of 8- to 12-week-old female and male immunodeficient NSG mice^16,28^. In brief, mice were anesthetized, and the right kidney was exposed. After a small incision was made into the renal capsule, it was carefully lifted from the functional tissue to allow for insertion of the organoid. At last, the implanted organoid was pushed an additional ~5 mm away from the incision. To test vascular connection of BMO transplants, mice were injected intravenously with 70 kDa FITC–dextran (1.25 mg in 50 µl per mouse, Invitrogen). Transplantations were carried out under the animal license number 2022-0.429.375 according to Austrian legislation. Transplants were performed with the help of the Preclinical Phenotyping Facility at Vienna BioCenter Core Facilities (VBCF)—a member of the Vienna BioCenter (VBC), Austria.

### Mouse xenotransplantation

NSG-HLA-DQ8 mice (3–4 weeks old) were preconditioned using 2.0 Gy X-ray irradiation (CP-160 Cabinet X-Radiator System). CD34^+^ sorted BMO-derived hematopoietic progenitor cells or CD34^+^ human CB cells (STEMCELL Technologies, catalog no. 7008.1) were injected intravenously (1.0 × 10^5^) into irradiated (2 Gy) NSG-HLA-DQ8 mice. To analyze reconstitution efficiency, peripheral blood samples were taken at 5, 8 and 10 weeks post transplantation and the presence of human hematopoietic cells was analyzed by flow cytometry. Mice were euthanized 10–12 weeks post transplantation and peripheral blood and BM cells were analyzed by flow cytometry.

### Artificial thymic organoid generation

The MS5-hDLL4 cell line was established as described previously^49^. In summary, MS5 cells (DMSZ, catalog no. ACC441) were transduced using a lentiviral vector encoding full-length human DLL4 cDNA. Subsequently, these cells were labeled with an anti-DLL4 antibody (Biolegend, catalog no. 346507, 1:50) and the population displaying the highest 5% DLL4 expression was isolated using FACS. Sorted cells were then cultured in DMEM/10% FCS. The stability of DLL4 expression was validated routinely through flow cytometry analysis. On the day of ATO generation, MS5-hDLL4 cells were harvested by trypsinization and resuspended in ATO medium, RPMI1640 (Gibco) supplemented with 4% B27 (Gibco), 30 µM l-ascorbic acid 2-phosphate sesquimagnesium salt (Sigma-Aldrich), 1% penicillin/streptomycin (Gibco), 1% Glutamax (Gibco), SCF (10 ng ml^−1^), Flt-3L (5 ng ml^−1^) and IL-7 (5 ng ml^−1^), all from STEMCELL Technologies. Per ATO, 1.5 × 10^5^ MS5-hDLL4 cells were coaggregated with 1 × 10^4^ BMO-derived sorted CD34^+^ cells and subsequently plated onto 0.4 mm Millicell transwell inserts (EMD Millipore). The inserts were placed in six-well plates, containing 1.5 ml of ATO medium per well. ATO medium was replaced completely every 3–4 days.

### Flow cytometry

Flow cytometry analysis was performed on day 10, 17, 21, 24, 45 and 60 of differentiation. For flow cytometry analysis, organoids were collected and washed once with PBS. Then, organoids were dissociated enzymatically into single cells with 3U ml^−1^ Dispase II (Gibco), 2U ml^−1^ Liberase (Roche) and 100U ml^−1^ DNAse (STEMCELL Technologies) in PBS for 25 min at 37 °C. During the incubation period, organoids were disrupted mechanically after 10, 20 and 25 min by vigorous pipetting using a P1000 and P200 pipette. After 25 min, the dissociation reaction was stopped and single cells were washed with FACS buffer (2% FBS in PBS) and collected by centrifugation at 300*g* for 5 min. Cells were resuspended in FACS buffer, strained through a 70 μm mesh, counted and incubated for 7 min with Fc block (Human True Stain FcX, Biolegend). Afterwards, cells were stained for 20 min in the dark at RT with flow cytometry antibodies listed in Supplementary Table 1. Antibody-labeled cells were washed with FACS buffer before proceeding to measuring. For the AnnexinV apoptosis detection assay, cells were washed with AnnexinV Binding Buffer (Invitrogen) and subsequently stained with fluorophore-conjugated antibodies and anti-AnnexinV. NSG mice with successful kidney transplants were euthanized 2 weeks, 3 months and 7 months after transplantation, and the femur and tibia were harvested. The intact bones were sterilized in 70% ethanol for 60 s before the bones were crushed in PBS. The BM was extracted from the crushed bones and passed through a 70 μm filter. Following filtering, red blood cells were lysed with the BD Pharm Lyse kit (BD Biosciences, catalog no. 555899) according to the manufacturer’s instructions. Properly lysed BM cells were subsequently stained with a mix of eBioscience Fixable Viability Dye eFluor 780 (Invitrogen, catalog no. 65-0865-14) and CD16/CD32 Fc blocking antibody (BD Biosciences, catalog no. 553141) in PBS for 20 min at 4 °C in the dark. After the first stain, cells were washed with FACS buffer and subsequently stained with antibodies listed in Supplementary Table 1. NSG-HLA-DQ8 mice were euthanized 10 weeks and 12 weeks after the transplantation. BM was harvested by flushing bones with PBS/2%FBS. Blood and BM were stained with antibodies listed in Supplementary Table 1 and red blood cells were lysed with the BD FACS lysing solution. Following two washes with PBS/2%FBS, blood and BM cells were filtered through a 35 µm strainer cap before analysis. ATOs were submerged in staining buffer (PBS/2% FBS/2 mM EDTA), disintegrated mechanically by gentle pipetting and passed through a 50 µm strainer. Cells were subsequently labeled with antibodies listed in Supplementary Table 1. Flow cytometry measurements were performed using a BD FACS LSR Fortessa cytometer. For FACS, a BD FACS Aria cytometer was used. BD FACS DIVA software (v.9.0.1) was used to collect flow cytometry data. Appropriate gating was determined using fluorescence minus one controls (see the corresponding Supplementary Figs. 1 and 6).

### Flow cytometry data analysis

Flow cytometry data were analyzed in FlowJO v.10.8. *t*-SNE analysis of flow cytometry data from three independent differentiations was performed using equal sampling of 35,000 cells from each FCS file, with 1,000 iterations, a perplexity of 30 and a learning rate of 7,350. The FlowSOM algorithm was used to create a self-organizing map with 100 clusters and 16 metaclusters. Resulting metaclusters were applied onto the *t*-SNE map.

### BMO dissociation for scRNA-seq

For scRNA-seq, ten randomly selected organoids on days 17, 21 and 24 were pooled for dissociation. Organoids were then dissociated enzymatically into single cells as described above for flow cytometry analysis. After 25 min of incubation in the enzyme mixture, 0.4% bovine serum albumin (BSA)/PBS solution was added to stop the dissociation reaction, centrifuged for 5 min at 300*g* at 4°C and washed again with 0.4% BSA/PBS. Next, the cell suspension was filtered through a 70 µm mesh and counted to determine the cell number and fraction of dead cells by trypan blue staining. Cell viability was greater than 90% and final cell concentration was adjusted to 1,000 cells µl^−1^.

### scRNA-seq library preparation and sequencing

The cell suspension was used immediately for scRNA-seq library preparation with a target recovery of 10,000 cells and loaded to a 10x Genomics Chromium Controller for Gel beads in emulsion generation and barcoding using the Chromium Single Cell 3′ Reagent Kit v.3.1 Dual Index (10x Genomics) according to the manufacturer’s instructions. Following cDNA synthesis, single-cell cDNA libraries were prepared. Quality control was conducted after cDNA amplification and library construction by Bioanalyzer analysis and Qubit measurement. Libraries were pooled and sequenced according to 10x Genomics recommendations on an Illumina NovaSeq6000 system with a target read depth of 50,000 reads per cell.

### scRNA-seq data analysis

Sequencing data were aligned and quantified using the Cell Ranger Single-Cell Software Suite against the GRCh38 human reference genome (Ensembl release v.106). For the purpose of quality control and for downstream analysis, transcriptomic profiles were filtered according to the thresholds detailed below^71^. First, barcodes with fewer than 400 detected genes, more than 40,000 counts in total or with an expression of mitochondrial genes exceeding 10% of the total number of gene counts were discarded. Second, for the removal of doublets, we ran Scrublet (v.0.2.3)^72^ on each sample individually and with default parameters, filtering transcriptomic profiles with a predicted doublet score exceeding 0.2. Further, gene expression profiles were normalized to a total count number of 10,000 before log1p transformation using Scanpy routines (v.1.9.1)^73^. For the purpose of dimensional reduction, neighborhood analyses and clustering, we identified the top 4,000 highly variable genes among all samples using the corresponding Scanpy function with default parameters and performed a principal component analysis, accounting for the top 50 principal components. Cell types were annotated with reference to marker genes from published human datasets. Characteristic genes for each cell type from a one-versus-rest comparison can be found in Supplementary Table 2.

### CFU assay

To determine the proliferation and differentiation ability of BMO-derived HSPCs, a CFU assay was conducted with FACS-sorted CD45^+^ CD11b^−^CD34^+^ cells. The assay was performed in triplicate according to the manufacturer’s protocol. Briefly, sorted cells were diluted in IMDM (Gibco) medium containing 1% penicillin-streptomycin. Diluted cells were added to complete MethoCult H4435 enriched medium (STEMCELL Technologies). A total of 1,000 cells were seeded per 35 mm dish and CFUs were counted on day 14 using an inverted light microscope. Correct assignment of colony lineage was verified by May–Gruenwald–Giemsa staining of sampled colonies.

### Trilineage MSPC differentiation assay

BMO-derived sorted MSPCs were seeded in six-well plates at a density of 4 × 10^3^ cells cm^−2^. For expansion, cells were cultured in MesenCult basal medium. When cells reached approximately 90% confluence, osteogenic and adipogenic differentiation was induced as followed: adipogenic differentiation was induced with MesenCult adipogenic differentiation medium, and medium was refreshed every 3 days. On day 28 of differentiation, cells were fixed in 10% formalin for 30 mins at RT, and lipid droplets were visualized by Oil-red-O staining. Osteogenic differentiation was induced using MesenCult osteogenic differentiation medium. Cells were maintained for 28 days, and medium was refreshed every 3–4 days. On day 28, cells were fixed in 10% formalin for 30 mins at RT and calcium deposits were visualized by Alizarin Red-S staining. A 3D pellet differentiation system was used for chondrogenic differentiation: 5 × 10^5^ MSPCs were resuspended in MesenCult-ACF chondrogenic differentiation medium and transferred to a 15 ml canonical tube. Cells were pelleted at 300*g* for 5 min and the lid was loosened to allow gas exchange. Pellets were maintained for 21 days, and medium was refreshed every 3 days. On day 21 chondrogenic pellets were fixed in 10% formalin for 30 min at RT. Preparation of paraffin slides and histological analysis with Alcian-blue stain was performed according to standard protocols. Medium and differentiation kits were purchased from STEMCELL Technologies.

### Modeling inflammation

To evaluate emergency granulopoeisis in the BMOs, organoids were placed in a 96-well round bottom plate in triplicate. LPS (1 µg ml^−1^, Sigma-Aldrich) or heat-killed *L.* *monocytogenes* (1 × 10^9^ ml^−1^, InvivoGen) were added into the cell culture medium for 4 h and 24 h. BMOs were harvested to assess emergency granulopoeisis using flow cytometry after 24 h, and supernatants were used to assess the production of G-CSF (R&D) and pro-inflammatory cytokines IL-6 (R&D) and IL-8 (R&D) at indicated timepoints. Enzyme-linked immunosorbent assays were performed according to manufacturer’s protocols.

### Immunofluorescence

Sprouted EBs of day 10 were fixed with 4% paraformaldehyde (PFA) for 20 min and organoids from day 17 onwards were washed with PBS and fixed with 4% PFA for 1 h at RT. Afterwards, they were permeabilized and incubated in blocking buffer (3% FBS, 1% BSA, 0.5% Triton X-100 and 0.5% Tween) for 2 h at RT on a shaker. Incubation with primary antibodies diluted in blocking buffer was done overnight at 4°C on a shaker. For immunofluorescence analysis of xenotransplants following dextran injection, kidneys were fixed in 10% neutral buffered formalin, cryo-embedded (Tissue-Tek OCT compound, Sakura) and cryosectioned into 14 µm sections. The following primary antibodies and dilutions were used: anti-CD31 (R&D Systems, catalog no. AF806, 1:20), anti-CD34 (Abcam, catalog no. ab81289, 1:100), anti-CD41 (Abcam, catalog no. ab134131, 1:200), anti-CD45 (Abcam, catalog no. ab10558, 1:350), anti-CD45 AlexaFluor 647 (Biolegend, catalog no. 304018, 1:20), anti-Collagen II (Abcam, catalog no. ab34712, 1:100), anti-Collagen IV (Sigma, catalog no. AB769, 1:200), anti-CXCL12/SDF-1 (R&D Systems, catalog no. MAB350, 1:30), anti-LepR AlexaFluor 647 (BD, catalog no. 564376,1:30), anti-MPO (Abcam, catalog no. ab25989, 1:250), anti-Nestin (R&D Systems, catalog no. MAB1259, 1:20), anti-NGFR/CD271 (Sigma, catalog no. HPA004765, 1:500), anti-PDGFRβ (Cell Signaling, catalog no. 3169 S, 1:100), anti-PRRX1 (Sigma, catalog no. ZRB2165, 1:50), anti-RUNX1/AML-1 (Cell Signaling, catalog no. 4336T, 1:200), anti S100A8/A9 (Abcam, catalog no. ab17050, 1:200) and anti-SMA (Abcam, catalog no. ab5694, 1:200). The BMOs were then washed three times with PBST (0.05% Tween in PBS) followed by a 2-h RT incubation with corresponding secondary antibodies diluted 1:300 in blocking buffer. Secondary antibodies from Invitrogen were: AlexaFluor 488 donkey anti-goat (catalog no. A11055), AlexaFluor 488 donkey anti-mouse (catalog no. A21202), AlexaFluor 488 donkey anti-rabbit (catalog no. A21200), AlexaFluor 488 donkey anti-sheep (catalog no. A11015), AlexaFluor 488 goat anti-rabbit (catalog no. A11034), AlexaFluor 568 donkey anti-rabbit (catalog no. A10042), AlexaFluor 594 goat anti-mouse (catalog no. A11032), AlexaFluor 594 goat anti-rabbit (catalog no. A11012) AlexaFluor 633 donkey anti-sheep (catalog no. A21100) and AlexaFluor 633 goat anti-mouse (catalog no. A21052). Finally, BMOs were stained with 4,6-diamidino-2-phenylindole for 15 min at RT and washed three times for 10 min with washing buffer. The stained BMOs were then mounted into wells of a prepared mounting system with fluorescence mounting medium (DAKO). This system was comprised of a 1.0 mm iSpacer (SunJin Laboratory) attached to a 22 × 50mm no. 1.5 coverslip, with double-sided tape on the top side. Once mounted, another coverslip was place on top and the edges were sealed. For optical clearing, BMOs were placed after immunolabeling into one well of the mounting set-up and 65 µl of RapiClear v.1.47 (SunJin Laboratory) was added. After 10 min, the top coverslip (22 × 50mm no. 1.5) was added, and light pressure was applied to seal the sample. Slides were stored in the dark at 4 °C until imaging. Each antibody staining was performed a minimum of three times in independent organoids. Secondary antibody controls were run in parallel to assess unspecific binding or background fluorescence signal.

### Confocal microscopy

Confocal laser scanning microscopy of organoids was performed with either an inverted Zeiss LSM800 microscope or an inverted Leica SP8 3x microscope. Overview images of organoids were acquired through tile scans using a ×20/0.8 M27 air objective (Zeiss) or a ×25/0.95 water immersion objective (Leica), utilizing optical zoom for enhanced visualization of cellular architecture. Specific cellular structures were imaged with a ×63/1.4 Oil DIC M27 objective (Zeiss) with immersion oil (Immersol 518F, Zeiss). All fluorescent targets were recorded in individual spectral channels and acquired in sequential mode. Spectral channel settings were chosen to optimize intensity while limiting bleed-through and background noise. A series of *x*-*y*-*z* images along large-tiled areas was acquired to represent whole organoids in reconstructed 3D *z*-projections. *Z*-stacks were sampled with a *x*-*y*-*z*-resolution for proper Nyquist sampling over a range of depths. All image acquisition was accomplished using Zeiss ZEN blue software v.2.6 or Leica Application Suite (LAS) X (v.3.8). Image processing was performed with FIJI, ZEN blue (v.2.6), LASX (v.3.8) and Imaris (v.9.7.2) software packages, with similar processing settings applied for respective negative controls.

### Two-photon microscopy

Two-photon-microscopy was performed at the Core Facility Bioimaging of the Biomedical Center of the LMU with a Leica SP8 MP DIVE microscope. The microscope has an upright DM8 stand and a motorized Scientifica stage. A FLUOTAR ×16/0.60 IMM objective was used with Leica immersion liquid Type G. A Spectra Physics Insight ×3 laser delivered 925, 1,045, 760 and 1,250 nm as excitation wavelengths. Recording was in a stack sequential manner, spectral detection of hybrid detectors in the non-descanned DIVE module was set to 500–550 nm, 570–610 nm, 400–480 nm and 650–700 nm. Images were recorded with 1,024 × 1,024 pixels with a pixel size of 750–900 nm (variable zoom) at 400 lines per second and a *z*-distance of 5 µm. *Z*-dimensions ranged from 515 µm to 845 µm and are indicated in figure legends. Image acquisition was accomplished using LASX (v.3.8). Image processing was performed with Imaris (v.9.7.2) software packages, with similar processing settings applied for respective negative controls.

### Image rendering and analysis

The 3D reconstruction and surface rendering of confocal and two-photon images were performed using the Imaris v.9.7.2. software package (Bitplane) with .ims as standard file format. Large confocal tiles scans were stitched with the Imaris v.9.7.2 Stitcher software (Bitplane) on computer at the Bioimaging core facility with 256 GB RAM, Intel Xeon CPU E5-2643 v.4 @ 3.4 GHz, 31-inch monitor with 4,096 × 2,160 pixels and a Nvidia Quadro RTX 5000 GPU with 16 GB GDDR6. Parallel-processing CUDA cores, 3,072; Tensor cores, 384; RT cores, 48.

### Angiotool

Three *z*-stacks of tile scans of whole-mount organoids were processed via the Angiotool interface^27^ to quantify total number of junctions, vessel area and branching index. Branching index is calculated as vessel area divided by junctions.

### Immunohistochemistry

Organoids were fixed with 4% PFA for 1 h at RT, washed with PBS and embedded in paraffin. Organoid sections of 1.5 µm thickness were generated using the Leica RM2245 microtome (Leica). Organoid sections were stained with H/E or Gomori using standard protocols or used for immunohistochemistry staining. After antigen retrieval, slides were incubated with CD34 (Sigma-Aldrich, catalog no. QEBnd-10; 1:100) or MPO (DAKO/Agilent, catalog no. A0298, 1:400) for 1 h. Detection was carried out with ImmPress anti-rabbit IgG polymer kit (Vector Laboratories) or MACH 3 Mouse HRP Polymer detection (Biocare Medical), respectively, according to the manufacturer´s protocol. Images were acquired on a Carl Zeiss Axioplan 2 or a Leica DM 2000 microscope. Reticulin staining was assessed by an experienced hematopathologist.

### Histology of xenotransplants

Mouse kidneys were harvested, cleared from residual fat tissue and fixed overnight by immersion in 10% neutral buffered formalin. They were processed with an automated tissue processor (Donatello I, DIAPATH), embedded in paraffin, sectioned at a thickness of 2 μm with a standard rotary microtome (Microm HM 355, Thermo Fisher Scientific), collected on glass slides and dried in the oven at 50 °C overnight. Sections were stained with H/E or hCD31 antibody (R&D, catalog no. AF806) on an automated staining platform (Epredia Gemini AS Automated Slide Stainer). Whole-slide images were prepared using the Pannoramic FLASH 250 III whole-slide scanner (3D Histech) with the ×40/0.95 plan apochromat objective and Adimec Quartz Q12A180 camera for bright-field and a pco.edge 4.2 4MP camera for fluorescent samples. Representative images were acquired from whole-slide images with the Case Viewer Software (3D Histech). Histology of xenotransplants was performed by the Histology Facility at VBCF.

### Cytospins

Target populations were FACS-sorted into Eppendorf tubes containing FACS buffer or picked colonies from the CFU assay were centrifuged in PBS for 10 min. Next, around 1 × 10^4^ cells were centrifuged at 500 rpm at RT for 5 min onto cytoslides (Tharmac) using a Cellspin I Cytocentrifuge (Tharmac) and air-dried overnight. Slides were stained with May–Gruenwald solution (Merck) for 2 min, followed by Giemsa azur-eosin-methylene blue solution (Merck) diluted 1:20 in Sorensen’s buffer for 17 min. Slides were viewed using a Leica DM 2000 microscope with a ×100 objective.

### Electron microscopy

Two randomly picked organoids were fixed using a mixture of 2% glutaraldehyde (Agar Scientific) and 2% PFA (Electron Microscopy Sciences) in 0.1 mol l^−1^ sodium cacodylate buffer, pH 7.2. for 4 h at RT. Fixative was then removed and organoids were stored in storage buffer at 4°C overnight. The next day, samples were rinsed three times for 10 min with the same buffer and postfixed in 2% osmium tetroxide (Agar Scientific) in 0.1 mol l^–1^ sodium cacodylate buffer. After another three washing steps the organoids were dehydrated in a graded series of acetone and embedded in Agar 100 resin (Agar Scientific). Subsequently, 70-nm sections were cut and poststained with 2% uranyl acetate and Reynolds lead citrate (Delta Microscopies). Micrographs of representative sections from two different organoids were recorded on an FEI Morgagni 268D (FEI) operated at 80 keV, equipped with a Mega View III CCD camera (Olympus-SIS) and in a Tecnai G2 20 operated at 200 keV (FEI), using an Eagle 4k HS camera. Electron microscopy studies were performed by the Electron Microscopy Facility at VBCF.

### Data visualization, statistical analysis and reproducibility

Data visualization and statistical analysis was done in GraphPad Prism v.9. Flow cytometry data were analyzed in FlowJo v.10. Confocal images were processed and analyzed in Fiji, ZEN blue edition v.2.6 and Imaris v.9.7.2. The data are presented as mean ± s.e.m. For statistical analysis, a two-tailed unpaired *t*-test or a two-way analysis of variance followed by Sidakʼs multiple comparison were performed. The differentiation protocol was replicated by five different investigators (S.F.-W., I.G., S.D.F., M.K. and P.C.) using iPSC1–5 and at least four different batches of Matrigel (lot numbers 0048007, 1013002, 1032003, 2067001) with similar results.

### Reporting summary

Further information on research design is available in the Nature Portfolio Reporting Summary linked to this article.

## Online content

Any methods, additional references, Nature Portfolio reporting summaries, source data, extended data, supplementary information, acknowledgements, peer review information; details of author contributions and competing interests; and statements of data and code availability are available at 10.1038/s41592-024-02172-2.

### Supplementary information

Supplementary Information Supplementary Note, Figs. 1–10 and Table 1.

Reporting Summary

Supplementary Table 2 Differentially expressed genes in scRNA-seq subclusters. The top differentially expressed genes in different scRNA-seq subclusters were identified through unequal variances *t*-tests comparing each subcluster against the rest. The tests were two-sided, and adjustments for multiple comparisons were not applied.

Supplementary Video 1 Spatial architecture of whole-mount organoid by two-photon microscopy.

Supplementary Video 2 Visualization of CD45^+^ cells by two-photon microscopy. Coloring based on distance to image border.

Supplementary Video 3 Spatial architecture of the BMO by immunostaining for CD31, CD45 and Nestin by two-photon microscopy.

Supplementary Video 4 3D surface rendering of CD31^+^ vascular structures in optically cleared BMO by confocal microscopy.

Supplementary Video 5 3D surface rendering of immunostaining for CD31, CD45 and CD271 visualizing CD45^+^ cells inside vessel lumen by confocal microscopy.

Supplementary Video 6 3D surface rendering of immunostaining for CD31, CD45 and Nestin visualizing CD45^+^ cells inside vessel lumen by confocal microscopy.

Supplementary Video 7 3D surface rendering of immunostaining for CD31, CD34 and CD45 inside a vessel lumen in the BMO at day 10 by confocal microscopy.

Supplementary Data 1 Source data for Supplementary Figures.

### Source data

Source Data Fig. 1 Statistical source data.

Source Data Fig. 3 Statistical source data.

Source Data Fig. 5 Statistical source data.

Source Data Fig. 6 Statistical source data.

Source Data Extended Data Fig. 1 Statistical source data.

Source Data Extended Data Fig. 2 Statistical source data.

Source Data Extended Data Fig. 3 Statistical source data.

Source Data Extended Data Fig. 4 Statistical source data.

Source Data Extended Data Fig. 5 Statistical source data.

## Data Availability

The scRNA-seq data presented in this study are available and can be accessed online for interactive exploration via the CellxGene portal explorer (cellxgene.cziscience.com). Raw and processed sequencing data are deposited at the Gene Expression Omnibus (GEO) repository under the accession code (GSE249005). Additionally, Scanpy h5ad objects of the preprocessed scRNA-seq data are available for download via the CellxGene portal explorer. Source data are provided with this paper.

## Code availability

No custom code was used for the analysis presented in this study. Single-cell sequencing data were processed and analyzed using publicly available software packages.
