# Supplementary material for: Generation of complex bone marrow organoids from human induced pluripotent stem cells
Source: Nat Methods. 2024 Feb 19;21(5):868–81. doi: 10.1038/s41592-024-02172-2 (PMC11093744; doi:10.1038/s41592-024-02172-2)
Supplement: Supplementary file 1 — Supplementary Note, Figs. 1–10 and Table 1. [file 41592_2024_2172_MOESM1_ESM.pdf]

nature methods

Article https://doi.org/10.1038/s41592-024-02172-2

Generation of complex bone marrow organoids from human induced pluripotent stem cells

In the format provided by the authors and unedited

Supplementary Figures 1-10, Supplementary Note and Supplementary Table 1

Supplementary Figure 1

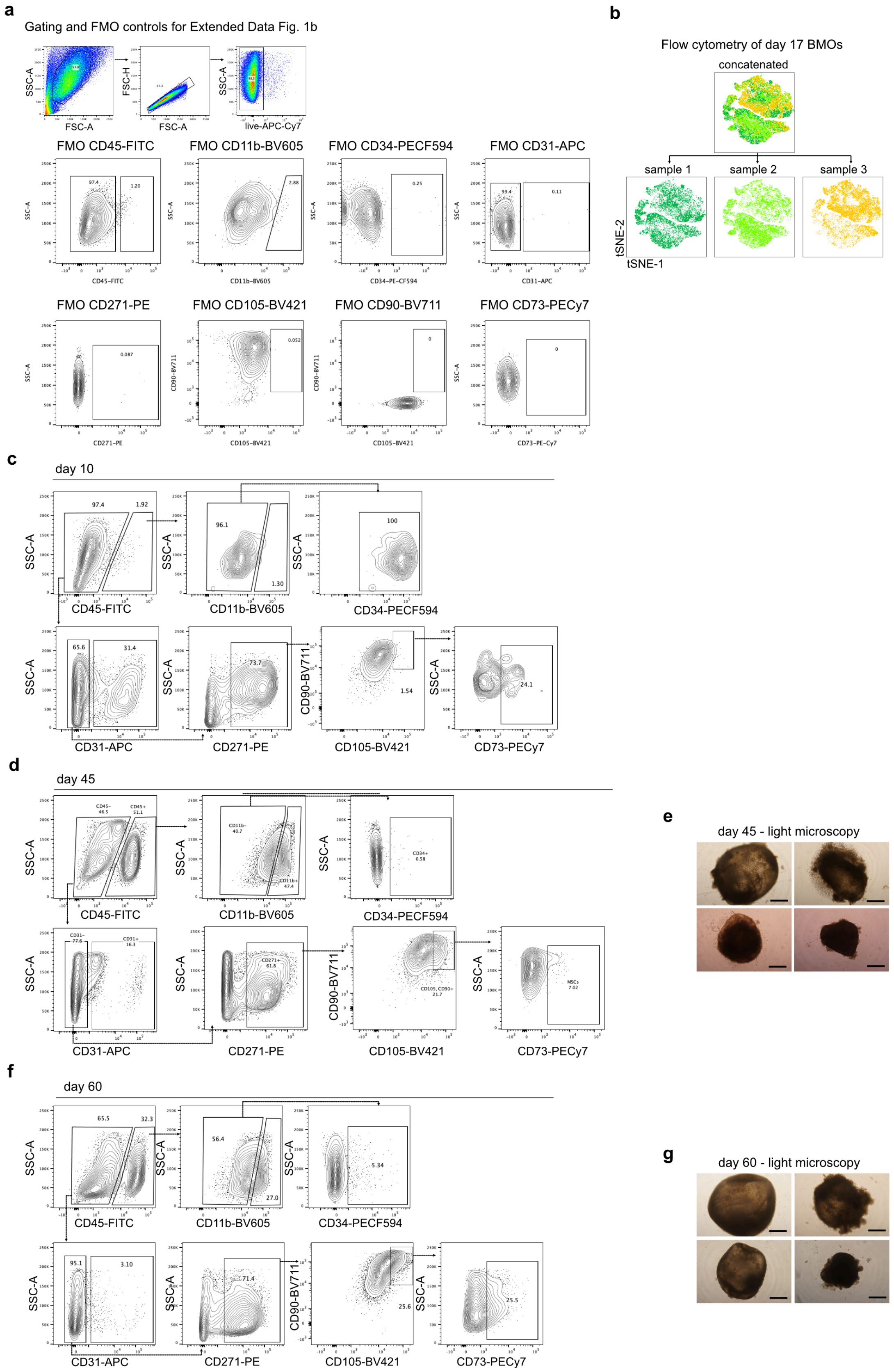

Supplementary Figure 1. **a)** Gating and plots of fluorescence minus one (FMO) controls for gating in Extended Data Fig. 1a. **b)** Joint concatenated tSNE visualization coloured by sample ID. Contribution of each data set shows negligible batch effects between three independent rounds of differentiation. **c)** Representative flow cytometry analysis plots of iPSC#1-BMOs on day 10 of differentiation. **d)** Representative flow cytometry analysis plots of iPSC#1-BMOs on day 45 of differentiation and **e)** corresponding bright field images. **f)** Representative flow cytometry analysis plots of iPSC#1-BMOs on day 60 of differentiation and **g)** corresponding bright field images. **c)-g)** *n*=3 independent differentiations. Scale bar in e) and g) 500µm.

Supplementary Note: To determine the reproducibility of the protocol, we generated BMOs by using additional iPS cell lines from different origins: two iPS cell lines derived from exfoliated renal epithelial cells present in urine, so-called urinary iPSCs (UiPSCs, iPSC#2 and #5)[1,2], as well as two commercially available cell lines, one PBMC-derived (SCTi-003A, iPS#3) and another fibroblast-derived iPS cell line (WTC-11, iPSC#4). The BMOs derived from iPSC#2-5 showed a highly similar morphology to iPSC#1 (Ext. Data Fig. 1d,e; mean diameter iPSC#2 1100µm, iPSC#3 880µm, iPSC#4 1124µm, iPSC#5 962 µm). While in all five iPS cell lines the main fractions, including hematopoietic cells ($CD45^+$), ECs ($CD45^-CD31^+$) and mesenchymal cells ($CD45^-CD31^-CD271^+$) could be identified within the BMOs, we observed a certain degree of variability in the proportions among different iPS cell lines (Ext. Data Fig. 1f, Supp. Fig. 2). Specifically, iPS cell lines #2, #3, and #4 contained a higher percentage of mesenchymal cells and a lower proportion of $CD45^+$ cells compared to iPSC#1 and iPSC#5 (Ext. Data Fig. 1f, Supp. Fig. 2). Notably, HSPCs ($CD45^+CD11b^-CD34^+$) and MSPCs ($CD45^-CD31^-CD271^+CD90^+CD105^+CD73^+$) were present in BMOs generated from all of the iPSC lines. The frequencies of these cell populations were comparable among the iPSC progenies, ranging from 1.10-2.81% for HSPCs and 0.52-1.36% for MSPCs (Ext. Data Fig. 1f, Supp. Fig. 2).

Supplementary Figure 2

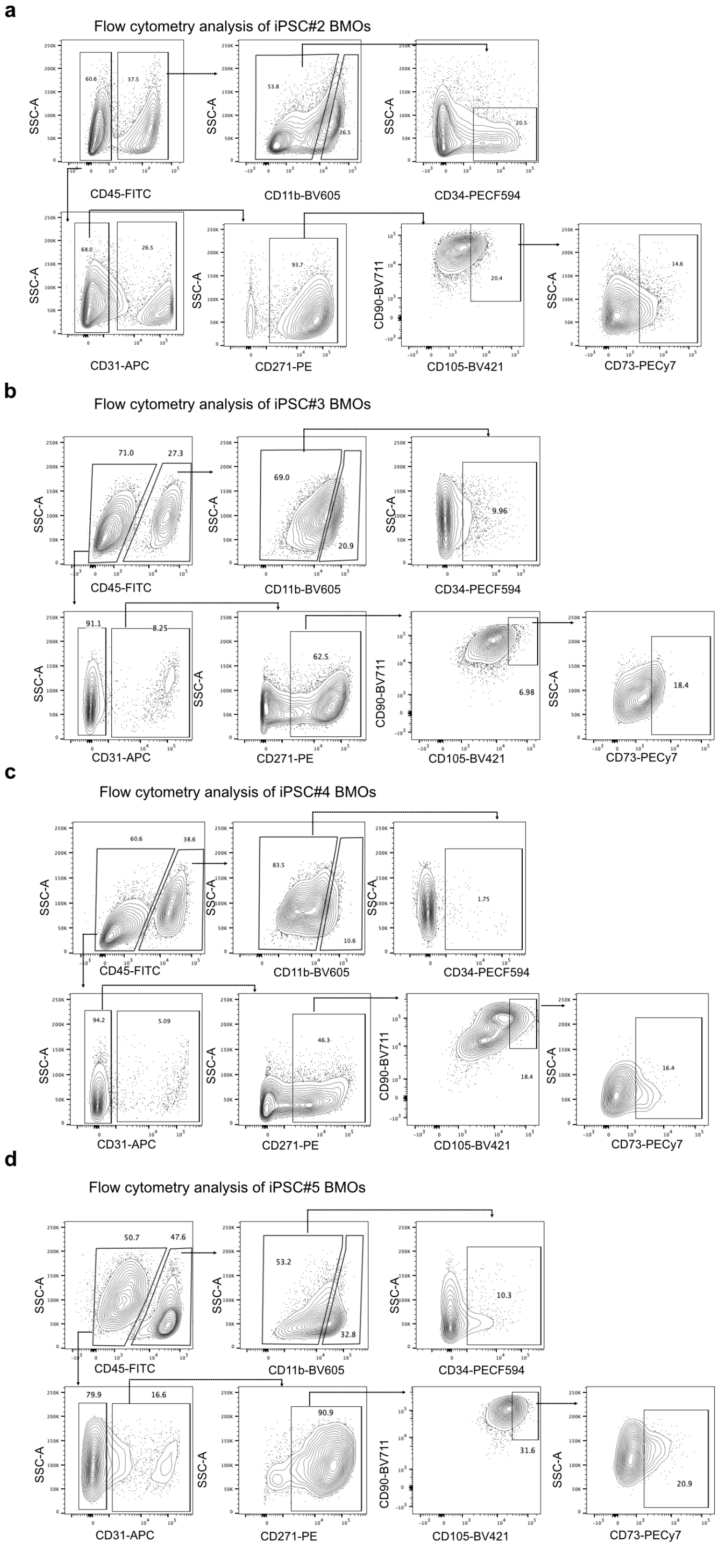

Supplementary Figure 2. Representative flow cytometry analysis plots of BMOs derived from different iPS cell lines between day 17-24 of differentiation, related to Ext. Data Fig. 1f, of **a)** iPSC#2-derived BMOs, **b)** iPSC#3-derived BMOs, **c)** iPSC#4-derived BMOs, **d)** iPSC#5-derived BMOs.

Supplementary Figure 3

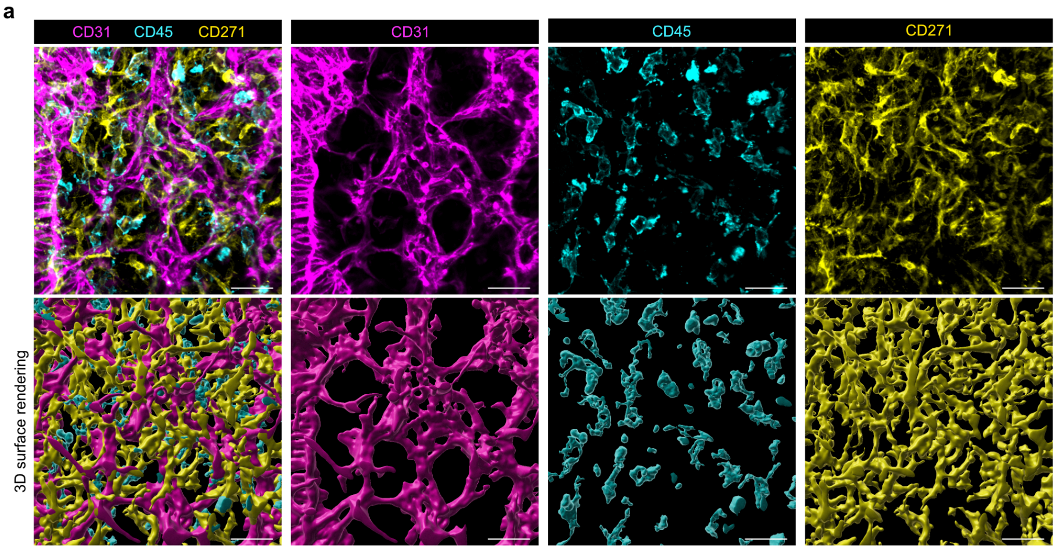

Supplementary Figure 3. a) Z-stack of confocal immunofluorescence images and corresponding 3D-surface rendering, related to Fig. 1 j), k) and Supp. Video 5. Scale bar 50µm.

Supplementary Figure 4

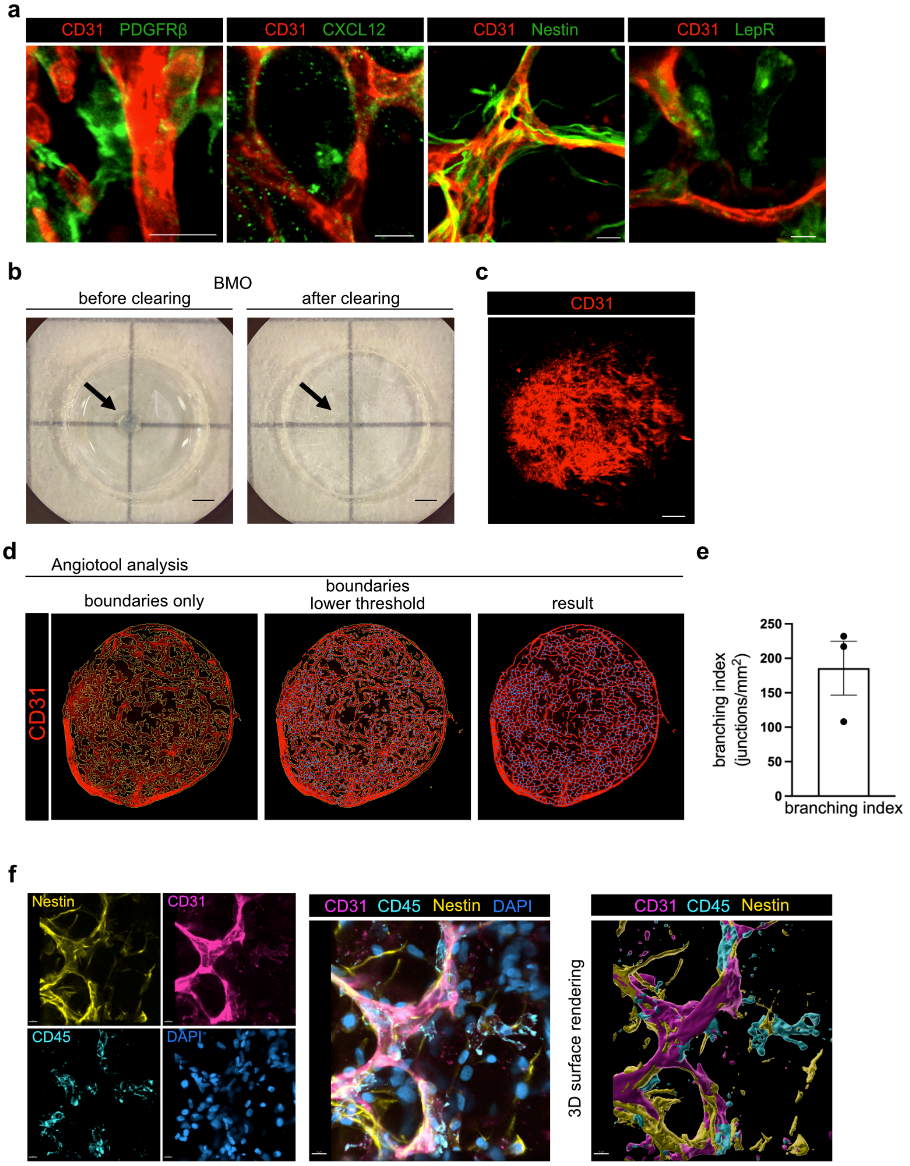

Supplementary Figure 4. a) Z-stack of confocal immunofluorescence images related to 3D-surface rendering in Fig. 2h). **b)** Brightfield images of BMO before and after clearing step, *n*=2 independent experiments. **c)** Z-stack of confocal immunofluorescence image of a cleared organoid related to 3D-surface rendering in Fig. 2i) and Supp. Video 3. **c)** Analysis of the workflow of maximum intensity projection of z-stack stained for CD31 computed with Angiotool. **d)** Branching index calculated as number of junctions per vessel area for *n*=3 independent experiments. Data are shown as mean ± SEM. **e)** Corresponding immunofluorescence confocal images of z-stack for 3D-surface rendering related to Ext. Data Fig. 3n). Scale bars in a), e) 10µm, b), 1mm, c) 100µm.

Supplementary Figure 5

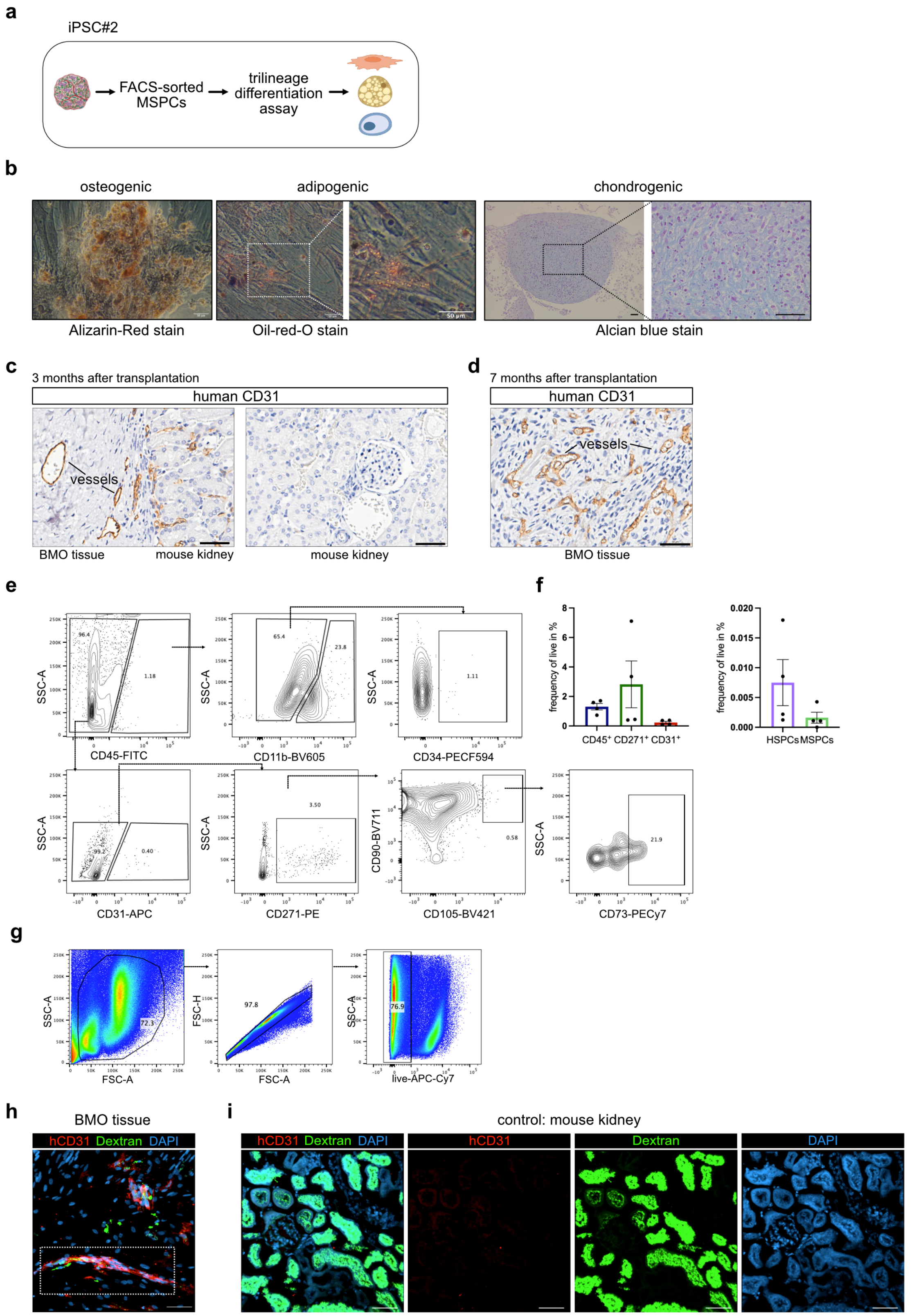

Supplementary Figure 5. a) Scheme for trilineage differentiation assay of sorted MSPCs from iPSC#2-derived BMOs. **b)** Osteogenic, adipogenic and chondrogenic differentiation visualized by Alizarin-Red-S, Oil-red-O or Alcian-Blue staining, *n*=2 for iPSC#2. **c)** Immunohistochemistry with human-specific CD31 antibody to determine BMO origin of vessels at 3

months. Right: mouse kidney tissue as negative control; *n*=3 xenotransplants. **d)** Immunohistochemistry with hCD31 antibody in BMO transplants after 7 months; *n*=2 xenotransplants. **e)** Representative plots of flow cytometry analysis of BMOs at 3 months after transplant. **f)** Quantification of frequencies of cell types of BMOs at 3 months after transplantation; *n*=4 xenotransplants. Data are presented as mean values $\pm$ SEM. **g)** Ancestry plots of mouse BM analysis for Ext. Data Fig. 4g. **h)** Immunofluorescence analysis of BMO transplants after intravenous injection of FITC-labelled dextran. Mouse kidney as negative control for hCD31 antibody and positive control for successful dextran injection as evidenced by dextran within the kidney cells; *n*=3 mice. Scale bars in b) 50µm, c),d) 20µm, h),i) 50µm.

Supplementary Figure 6

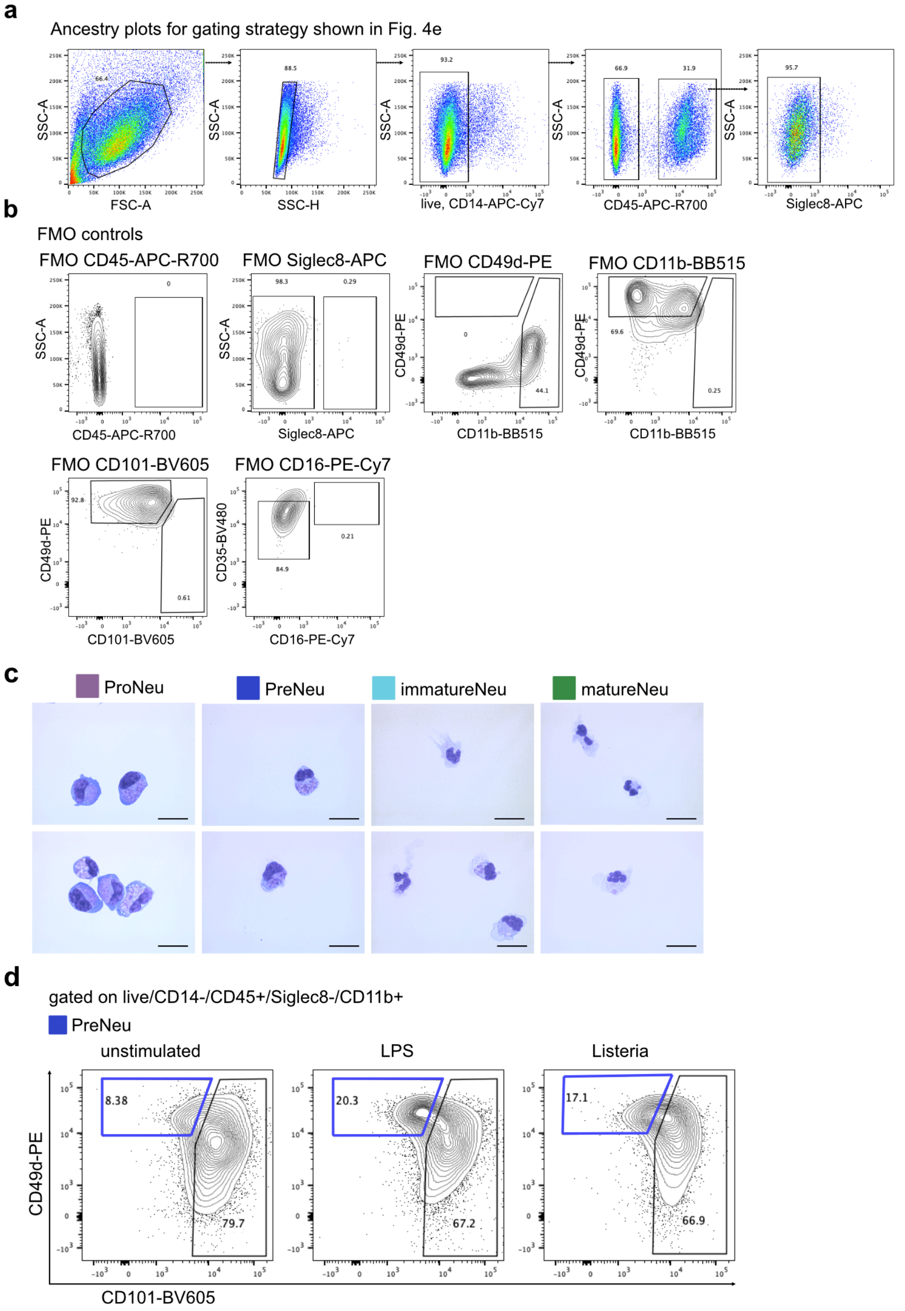

Supplementary Figure 6. a) Ancestry plots for gating strategy shown in Figure 3 e). **b)** FMO-controls for gating strategy in Fig. 3 e. **c)** Morphology of sorted and May-Gruenwald-Giemsa-stained neutrophil progenitor populations. Representative images from *n*=2 independent experiments. Scale bar in c) 20µm. **d)** Representative flow cytometry plots for Fig. 3 k).

Supplementary Figure 7

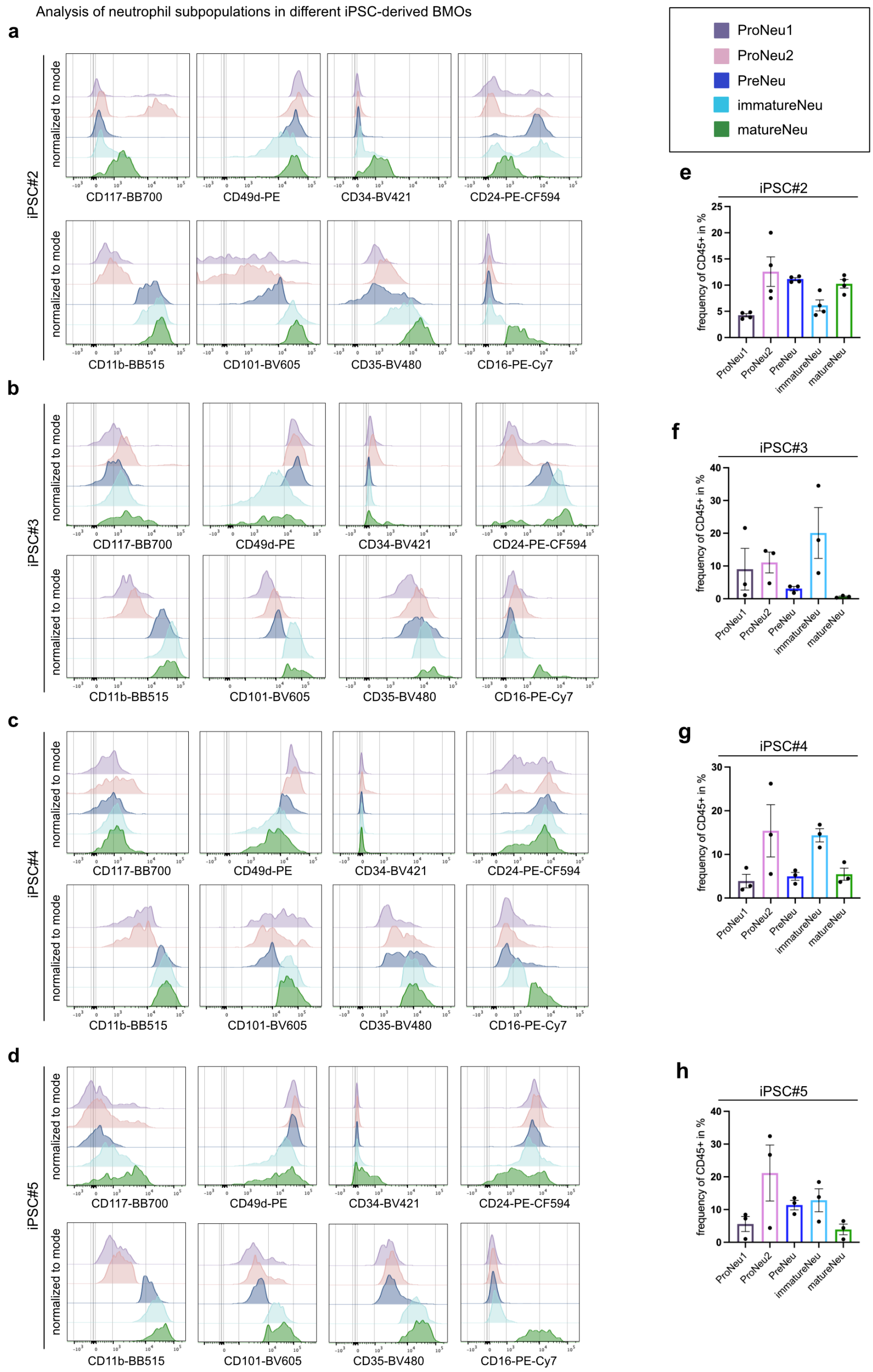

Supplementary Figure 7. **a-d)** Representative histograms of flow-cytometric analysis of iPSC#2-#5derived BMOs. **e-h)** Frequencies of different progenitor stages; **e)** *n*=4 independent differentiations, **f)-h)** *n*=3 independent differentiations. Data in **e-h)** are presented as mean values ± SEM.

Supplementary Figure 8

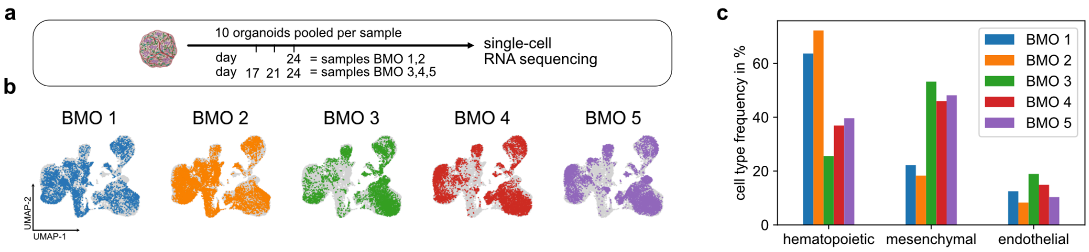

Supplementary Figure 8. a) Experimental outline for scRNA-seq analyses. 10 iPSC#1-derived organoids were pooled per time point on day 17, 21 and 24 of differentiation (3 samples). In a second independent batch of differentiation two samples of organoids on day 24 were analyzed. **b)** Contributions of individual samples to UMAP plot shown in Figure 4. **c)** Frequency distribution of main cell populations defined by scRNA-seq per sample.

Supplementary Figure 9

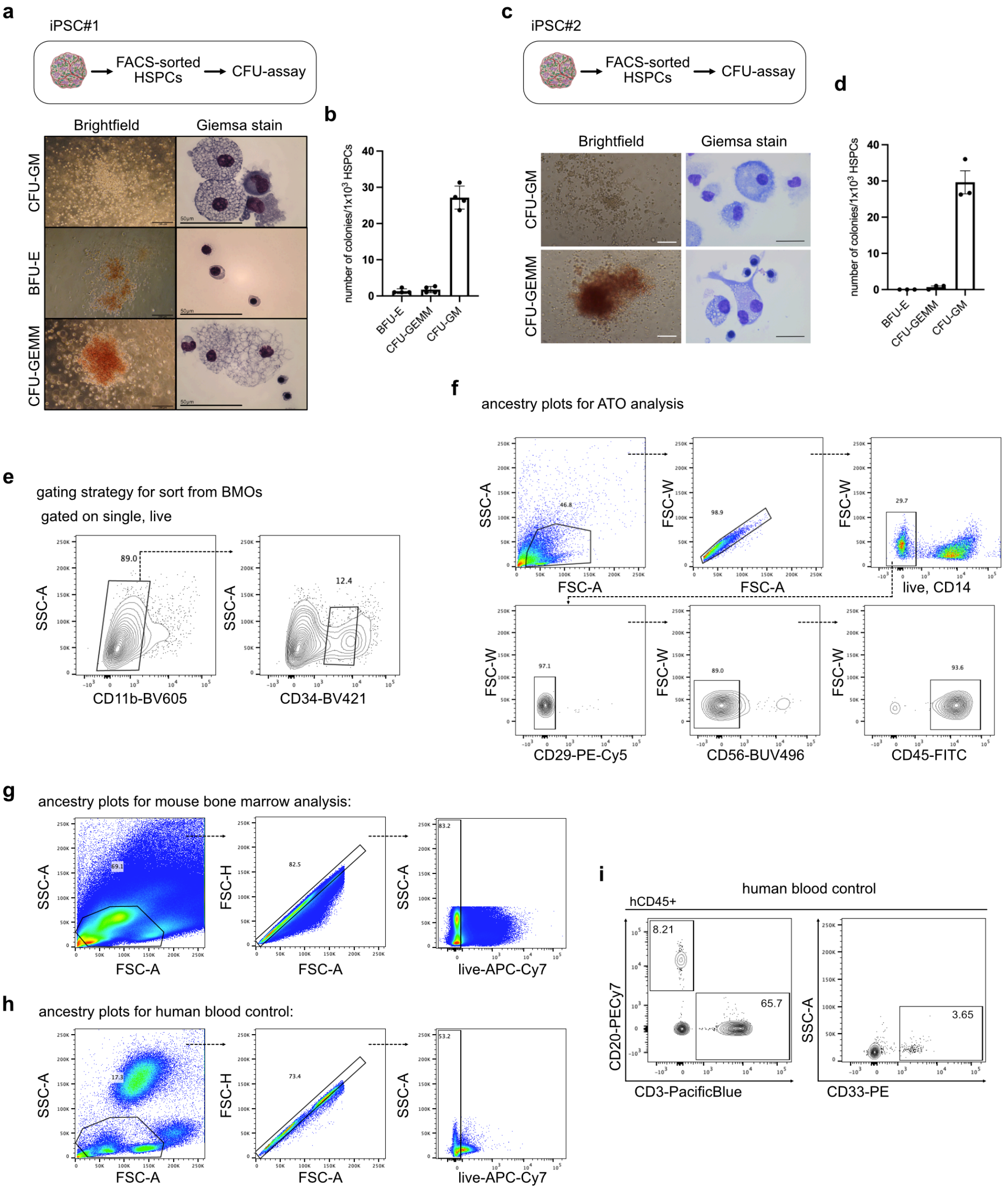

Supplementary Figure 9. Colony-forming unit assay of FACS-sorted BMO-derived $CD45^+CD11b^-CD34^+$ HSPCs from **a)** iPSC#1 and **c)** iPSC#2. CFU-GM, granulocyte-macrophage colony-forming unit, BFU-E, erythroid burst-forming unit, CFU-GEMM, granulocyte, erythrocyte, monocyte, megakaryocyte colony-forming unit; Representative phase contrast microscopy of colony morphology (left) and May-Gruenwald-Giemsa stain of picked colonies (right). **b), d)** Quantification of frequencies of BMO HPSC-derived colonies, *n*=4 independent experiments for iPSC#1 and *n*=3 independent experiments for iPSC#2. Data in **b)** and **d)** are presented as mean values ± SEM. **e)** Gating strategy for sorting single, live, $CD11b^-$, $CD34^+$ cells. $CD34^{high}$ cells were excluded. **f)** Ancestry plots for Fig. 5e. **g), h)** Ancestry plots for Fig. 5 i. **i)** expression of CD20, CD33 and CD3 on human blood as control for Fig. 5k). Scale bars in a), c) 200µm (left) and 50µm (right).

Supplementary Figure 10

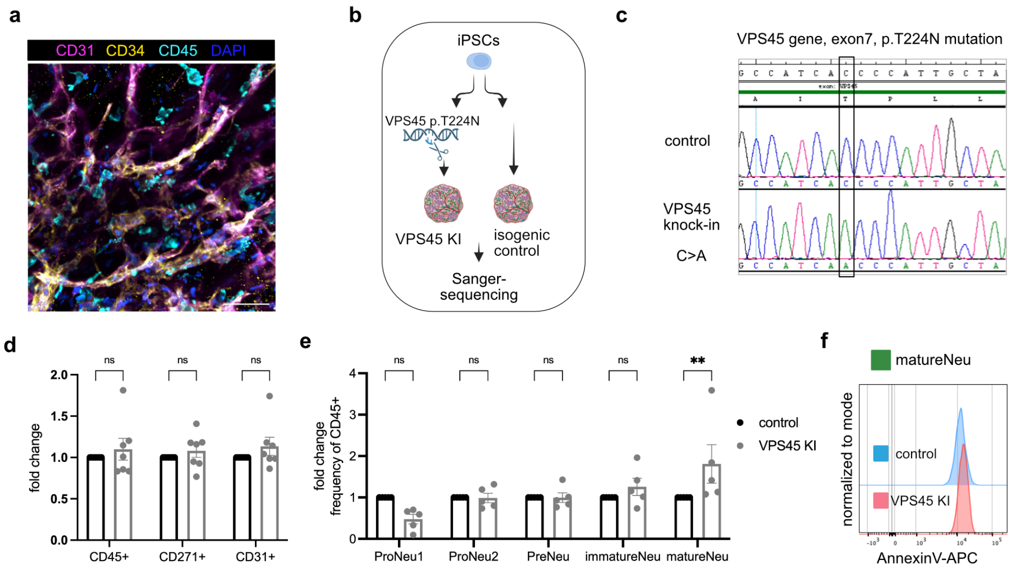

Supplementary Figure 10. a) Immunostaining of CD31, CD34 and CD45 in BMOs at day 10 by confocal imaging. Related to Fig. 6k. Scale bar 50µm, *n*=3 independent experiments. **b)** Schematic overview of the gene-editing approach to generate the isogenic VPS45-mutant iPS cell line with isogenic background and **c)** Sanger-sequencing of the p.T224N mutation before start of each differentiation. **d)** Comparison of main cell type frequencies between control and VPS45 mutant BMOs by flow cytometry, *n*=7 independent experiments; two-way ANOVA followed by Sidaks multiple comparisons test. **e)** Comparison of neutrophil subset frequencies of $CD45^+$ cells between control and VPS45 mutant BMOs by flow cytometry, *n*=5 independent experiments; **p=0.0096, two-way ANOVA followed by Sidaks multiple comparisons test. Data in **d)** and **e)** are presented as mean values ± SEM. **f)** Representative histogram of Annexin V expression intensity on matureNeu subsets in control and VPS45 mutant BMOs, *n*=3 independent experiments.

Supplementary Table 1. Flow cytometry antibodies.

Marker	Fluorochrome	Clone	Cat. Nr.	Company
CD3	Pacific Blue	SK7	344824	BioLegend
CD3	BV650	UCHT1	563852	BD
CD4	BV480	RPA-T4	746541	BD
CD5	APC	UCHT2	555355	BD
CD7	BB700	M-T701	566488	BD
CD8	PE	QA18A37	303804	BioLegend
CD10	PE	HI10A	555375	BD
CD11b	BV605	ICRF44	562721	BD
CD11b	BB515	ICRF44	564517	BD
CD14	APC-Fire 750	M5E2	301854	BioLegend
CD16	PE-Cy7	3G8	557744	BD
CD19	R718	SJ25C1	566946	BD
CD20	PE-Cy7	2H7	560735	BD
CD24	PE-Dazzle 594	ML5	311134	BioLegend
CD31	APC	WM59	303116	BioLegend
CD31	PE-Cy7	WM59	303118	BioLegend
CD33	PE	HIM3-4	12-0339-42	Invitrogen
CD34	PE-Dazzle 594	581	343534	BioLegend
CD34	BV421	581	562577	BD
CD35	BV480	E11	746503	BD
CD41	PE	HIP8	557297	BD
CD42	APC	HIP1	551061	BD
CD45	FITC	HI30	304006	BioLegend
CD45	APC-R700	HI30	566041	BD
CD45	APC	HI30	304012	BioLegend
CD49d	PE	9F10	555503	BD
CD49d	BV421	9F10	304322	BioLegend
CD56	BUV496	NCAM16.2	750479	BD
CD61	FITC	VI-PL2	555753	BD
CD73	PE-Cy7	AD2	561258	BD
CD90	BV711	5E10	328140	BioLegend
CD101	BV605	V7.1	747548	BD
CD105	BV421	SN6h	800510	BioLegend
CD117	BB700	YB5.B8	566548	BD
CD127 (IL7R)	APC	A019D5	351316	BioLegend
CD184 (CXCR4)	BV480	12G5	746621	BioLegend
CD271	PE	ME20.4	345106	BioLegend
CD295 (LepR)	AF-647	52263	564376	BD
DLL4	APC	MHD4-46	346507	BioLegend
Siglec8	APC	7C9	347106	BioLegend
TCRab	PE-Cy7	IP26	306720	BioLegend

TCRcd	BV421	B1	562560	BD
mCD29	PE-Cy5	HMb1-1	102219	BioLegend
mCD45	FITC	30-F11	103108	BioLegend
mCD45	PE	30-F11	12-0451-83	Thermo Fisher
Annexin V	APC		17-8007-74	Thermo Fisher
Fixable viability stain	Red 780		565388	BD

Supplementary References:

1. Zhou, T. *et al.* Generation of human induced pluripotent stem cells from urine samples. *Nat. Protoc.* **7**, 2080–2089 (2012).
2. Geuder, J. *et al.* A non-invasive method to generate induced pluripotent stem cells from primate urine. *Sci. Rep.* **11**, 3516 (2021).
