# Supplementary material for: Generation of complex bone marrow organoids from human induced pluripotent stem cells
Source: Nat Methods. 2024 Feb 19;21(5):868–81. doi: 10.1038/s41592-024-02172-2 (PMC11093744; doi:10.1038/s41592-024-02172-2)
Supplement: Supplementary file 2 — Reporting Summary [file 41592_2024_2172_MOESM2_ESM.pdf]

nature portfolio

Corresponding author(s): Christoph Klein

Last updated by author(s): Dec 2, 2023

Reporting Summary

Nature Portfolio wishes to improve the reproducibility of the work that we publish. This form provides structure for consistency and transparency in reporting. For further information on Nature Portfolio policies, see our Editorial Policies and the Editorial Policy Checklist.

Statistics

For all statistical analyses, confirm that the following items are present in the figure legend, table legend, main text, or Methods section.

n/a	Confirmed	
☐	☒	The exact sample size (*n*) for each experimental group/condition, given as a discrete number and unit of measurement
☐	☒	A statement on whether measurements were taken from distinct samples or whether the same sample was measured repeatedly
☐	☒	The statistical test(s) used AND whether they are one- or two-sided<br>*Only common tests should be described solely by name; describe more complex techniques in the Methods section.*
☐	☒	A description of all covariates tested
☐	☒	A description of any assumptions or corrections, such as tests of normality and adjustment for multiple comparisons
☐	☒	A full description of the statistical parameters including central tendency (e.g. means) or other basic estimates (e.g. regression coefficient) AND variation (e.g. standard deviation) or associated estimates of uncertainty (e.g. confidence intervals)
☐	☒	For null hypothesis testing, the test statistic (e.g. *F*, *t*, *r*) with confidence intervals, effect sizes, degrees of freedom and *P* value noted<br>*Give P values as exact values whenever suitable.*
☒	☐	For Bayesian analysis, information on the choice of priors and Markov chain Monte Carlo settings
☒	☐	For hierarchical and complex designs, identification of the appropriate level for tests and full reporting of outcomes
☒	☐	Estimates of effect sizes (e.g. Cohen's *d*, Pearson's *r*), indicating how they were calculated

Our web collection on statistics for biologists contains articles on many of the points above.

Software and code

Policy information about availability of computer code

Data collection	ZEN v2.6. blue edition software (Zeiss) and Leica Application Suite (LAS) X (Leica) software was used for confocal microscopy, Leica Application Suite (LAS) X (Leica) software was used for recording of two-photon microscopy studies.<br>FACS DIVA software v9.0.1 (BD) was used to collect flow cytometry data.
Data analysis	Data visualization and statistical analysis was done in GraphPad Prism v9. Flow cytometry data were analysed in FlowJo v10. Confocal images were processed and analysed in Fiji, ZEN v2.6. blue edition and Imaris v9.7.2 (Bitplane). Two-photon images were processed and analysed in Imaris v9.7.2 (Bitplane).<br>The Scanpy package (version 1.9.1) was used for the analysis of scRNAseq data. Scrublet (version 0.2.3) was used for the removal of doublets. Any data filtering steps are detailed in the manuscript. Scanpy h5ad objects of the preprocessed scRNAseq data is available for exploration and download via cellxgene.cziscience.com.<br>Code Availability statement:<br>No custom code was used for the analysis presented in this study. Single-cell sequencing data were processed and analysed using publicly available software packages.

For manuscripts utilizing custom algorithms or software that are central to the research but not yet described in published literature, software must be made available to editors and reviewers. We strongly encourage code deposition in a community repository (e.g. GitHub). See the Nature Portfolio guidelines for submitting code & software for further information.

Data

Policy information about availability of data

All manuscripts must include a data availability statement. This statement should provide the following information, where applicable:

- Accession codes, unique identifiers, or web links for publicly available datasets
- A description of any restrictions on data availability
- For clinical datasets or third party data, please ensure that the statement adheres to our policy

The single cell RNA sequencing data presented in this study are available and can be accessed online for interactive exploration via the CellxGene portal explorer (cellxgene.cziscience.com). Raw and processed sequencing data are deposited at Gene Expression Omnibus (GEO) repository under the accession code GSE249005. Additionally, Scanpy h5ad objects of the preprocessed scRNAseq data are available for download via the CellxGene portal explorer.

Human research participants

Policy information about studies involving human research participants and Sex and Gender in Research.

Reporting on sex and gender	Two human iPSC lines, generated from urin samples were used, one male and one female.
Population characteristics	Students from Munich, Germany.
Recruitment	Students were recruited from graduate and under-graduate students of biology in Munich, Germany. Informed consent was obtained from all volunteers and urine samples were processed anonymously, but separately for males and females.
Ethics oversight	Ethikkommission Ludwig-Maximilians-University Munich, Germany (20-122).

Note that full information on the approval of the study protocol must also be provided in the manuscript.

Field-specific reporting

Please select the one below that is the best fit for your research. If you are not sure, read the appropriate sections before making your selection.

☒ Life sciences ☐ Behavioural & social sciences ☐ Ecological, evolutionary & environmental sciences

For a reference copy of the document with all sections, see nature.com/documents/nr-reporting-summary-flat.pdf

Life sciences study design

All studies must disclose on these points even when the disclosure is negative.

Sample size	No statistical methods were used to predetermine sample size. Samples sizes were based on determining sufficient replication to account for biological variation. For statistical analysis between different treatment groups or genotypes at least three different biological replicates were analyzed.
Data exclusions	No data were excluded in this study except standard removal of low quality single cells for downstream analysis of scRNA-seq data as described in the Methods section.
Replication	All experiments in this study were independently biologically replicated and all attempts of replication were successful.<br>The differentiation protocol was replicated by five different investigators (S.F.-W., I.G., S.D.F., M.K., P.C.) using five different iPS cell lines (iPSC#1-#5) and at least four different batches of Matrigel (lot numbers: 0048007, 1013002, 1032003, 2067001) with similar results.
Randomization	Organoids were randomly selected from organoid batches for subsequent expriments.
Blinding	Investigators were not blinded during experiments. Blinding was not applicable, since the same investigators performed data collection and analysis.

Reporting for specific materials, systems and methods

We require information from authors about some types of materials, experimental systems and methods used in many studies. Here, indicate whether each material, system or method listed is relevant to your study. If you are not sure if a list item applies to your research, read the appropriate section before selecting a response.

Materials & experimental systems

n/a	Involved in the study
☐	☒ Antibodies
☐	☒ Eukaryotic cell lines
☒	☐ Palaeontology and archaeology
☐	☒ Animals and other organisms
☒	☐ Clinical data
☒	☐ Dual use research of concern

Methods

n/a	Involved in the study
☒	☐ ChIP-seq
☐	☒ Flow cytometry
☒	☐ MRI-based neuroimaging

Antibodies

Antibodies used

Antibodies used for flow cytometry are included in Supplementary Table 1 and listed below.
Antibody, fluorophore, clone, catalog number, manufacturer, dilution (all antibodies anti-human, except m indicates anti-mouse):
CD3 PacificBlue SK7 344824 BioLegend 1:100
CD3 BV650 UCHT1 563852 BD 1:100
CD4 BV480 RPA-T4 7456541 BD 1:100
CD5 APC UCHT2 555355 BD 1:50
CD7 BB700 M-T701 566488 BD 1:100
CD8 PE QA18A37 303804 BioLegend 1:100
CD10 PE HI10A 555375 BD 1:30
CD11b BV605 ICRF44 562721 BD 1:100
CD11b BB515 ICRF44 564517 BD 1:100
CD14 APC-Fire 750 M5E2 301854 BioLegend 1:100
CD16 PE-Cy7 3G8 557744 BD 1:100
CD19 R718 SJ25C1 566946 BD 1:50
CD20 PE-Cy7 2H7 560735 BD 1:100
CD24 PE-Dazzle 594 ML5 311134 BioLegend 1:100
CD31 APC WM59 303116 BioLegend 1:100
CD31 PE-Cy7 WM59 303118 BioLegend 1:100
CD33 PE HIM3-4 12-0339-42 Invitrogen 1:100
CD34 PE-Dazzle 594 581 343534 BioLegend 1:100
CD34 BV421 581 562577 BD 1:100
CD35 BV480 E11 746503 BD 1:100
CD41 PE HIP8 557297 BD 1:30
CD42 APC HIP1 551061 BD 1:30
CD45 FITC HI30 304006 BioLegend 1:50
CD45 APC-R700 HI30 566041 BD 1:100
CD45 APC HI30 304012 BioLegend 1:50
CD49d PE 9F10 555503 BD 1:100
CD49d BV421 9F10 304322 BioLegend 1:100
CD56 BUV496 NCAM16.2 750479 BD 1:100
CD61 FITC VIPL2 555753 BD 1:30
CD73 PE-Cy7 AD2 561258 BD 1:100
CD90 BV711 5E10 328140 BioLegend 1:100
CD101 BV605 V7.1 747548 BD 1:100
CD105 BV421 SN6h 800510 BioLegend 1:100
CD117 BB700 YB5.B8 566548 BD 1:30
CD127 (IL7R) APC A019D5 351316 BioLegend 1:30
CD184 (CXCR4) BV480 12G5 746621 BD 1:50
CD271 PE ME20.4 345106 BioLegend 1:300
CD295 (LepR) AF647 52263 564376 BD 1:50
DLL4 APC MHD4-46 346507 BioLegend 1:50
Siglec8 APC 7C9 347106 BioLegend 1:100
TCRab PE-Cy7 IP26 306720 BioLegend 1:50
TCRcd BV421 B1 562560 BD 1:30
mCD29 PE-Cy5 HMb1-1 102219 BioLegend 1:100
mCD45 FITC 30-F11 103108 BioLegend 1:100
mCD45 PE 30-F11 12-0451-83 Thermo Fisher 1:100
Annexin V APC 17-8007-74 Thermo Fisher 1:50
Fixable viability stain Red780 565388 BD 1:400

The following anti-human primary antibodies and dilutions were used for immunofluorescence:
anti-CD31/PECAM1 (R&D Systems AF806, 1:20)
anti-CD34 (abcam ab81289, clone EP373Y, 1:100)
anti-CD41 (abcam ab134131, clone EPR4330, 1:200)
anti-CD45 (abcam ab10558, 1:350)
anti-CD45 Alexa Fluor 647 (Biolegend 304018, clone HI30, 1:15)
anti-Collagen II (abcam, ab34712, 1:100)
anti-Collagen IV (Sigma AB769, 1:200)
anti-CXCL12/SDF-1 (R&D Systems MAB350, clone 79018, 1:30)

anti-LepR Alexa Fluor 647 (BD, 564376, clone 52263, 1:30)
anti-MPO (abcam ab25989, clone 2C7, 1:250)
anti-Nestin (R&D Systems MAB1259, clone 196908, 1:20)
anti-NGFR/CD271 (Sigma HPA004765, 1:500)
anti-PDGFRβ (Cell Signaling 3169S, clone 28E1, 1:100)
anti-PRRX1 (Sigma, ZRB2165, 1:50)
anti-RUNX1/AML-1 (Cell Signaling 4336T, clone D33G6, 1:200)
anti S100A8/A9 (abcam ab17050, clone 27E10, 1:200)
anti-SMA (abcam ab5694, 1:200).

Secondary antibodies from Invitrogen were diluted 1:300: AlexaFluor 488 donkey anti-goat (A11055), AlexaFluor 488 donkey anti-mouse (A21202), AlexaFluor 488 donkey anti-rabbit (A21200), AlexaFluor 488 donkey anti-sheep (A11015), AlexaFluor 488 goat anti-rabbit (A11034), AlexaFluor 568 donkey anti-rabbit (A10042), AlexaFluor 594 goat anti-mouse (A11032), AlexaFluor 594 goat anti-rabbit (A11012) AlexaFluor 633 donkey anti-sheep (A21100), AlexaFluor 633 goat anti-mouse (A21052).

For immunohistochemistry the following antibodies were used: CD34 (Sigma-Aldrich, QEBnd-10; 1:100) or MPO (DAKO/Agilent, A0298, 1:400).

Validation

All antibodies used in this study are commercially available and have been validated for the application by the manufacturer or have been previously validated in other publications:
anti-CD31/PECAM1 (R&D Systems AF806, 1:20), https://www.rndsystems.com/products/human-cd31-pecam-1-antibody_af806
anti-CD34 (abcam ab81289, clone EP373Y, 1:100), https://www.abcam.com/products/primary-antibodies/cd34-antibody-ep373y-ab81289.html
anti-CD41 (abcam ab134131, clone EPR4330, 1:200), https://www.abcam.com/products/primary-antibodies/cd41-antibody-epr4330-ab134131.html
anti-CD45 (abcam ab10558, 1:350), https://www.abcam.com/products/primary-antibodies/cd45-antibody-ab10558.html
anti-CD45 Alexa Fluor 647 (Biolegend 304018, clone HI30, 1:15)
anti-Collagen II (abcam, ab34712, 1:100), https://www.abcam.com/products/primary-antibodies/collagen-ii-antibody-ab34712.html
anti-Collagen IV (Sigma AB769, 1:200), https://www.sigmaaldrich.com/DE/de/product/mm/ab769
anti-CXCL12/SDF-1 (R&D Systems MAB350, clone 79018, 1:30), https://www.rndsystems.com/products/human-mouse-cxcl12-sdf-1-antibody-79018_mab350
anti-LepR Alexa Fluor 647 (BD, 564376, clone 52263, 1:30), https://www.bdbiosciences.com/en-eu/products/reagents/flow-cytometry-reagents/research-reagents/single-color-antibodies-ruo/alexa-fluor-647-mouse-anti-human-leptin-receptor-cd295.564376
anti-MPO (abcam ab25989, clone 2C7, 1:250), https://www.abcam.com/products/primary-antibodies/myeloperoxidase-antibody-2c7-ab25989.html; used for IF of human neutrophils e.g. in Neubert et al., Nat. Communications, 2018.
anti-Nestin (R&D Systems MAB1259, clone 196908, 1:20), https://www.rndsystems.com/products/human-nestin-antibody-196908_mab1259
anti-NGFR/CD271 (Sigma HPA004765, 1:500), https://www.sigmaaldrich.com/DE/de/product/sigma/hpa004765
anti-PDGFRβ (Cell Signaling 3169S, clone 28E1, 1:100), https://www.cellsignal.com/products/primary-antibodies/pdgf-receptor-b-28e1-rabbit-mab/3169
anti-PRRX1 (Sigma, ZRB2165, 1:50), https://www.sigmaaldrich.com/DE/de/product/sigma/zrb2165
anti-RUNX1/AML-1 (Cell Signaling 4336T, clone D33G6, 1:200), https://www.cellsignal.com/products/primary-antibodies/aml1-d33g6-xp-rabbit-mab/4336
anti S100A8/A9 (abcam ab17050, clone 27E10, 1:200), https://www.abcam.com/products/primary-antibodies/s100a8--s100a9-antibody-27e10-ab17050.html;
anti-SMA (abcam ab5694, 1:200), https://www.abcam.com/products/primary-antibodies/alpha-smooth-muscle-actin-antibody-ab5694.html; used for IF in Wimmer et al., Nature, 2019

Eukaryotic cell lines

Policy information about cell lines and Sex and Gender in Research

Cell line source(s)

The human fibroblast-derived iPS cell line HMGU1 (iPSC#1) was provided by the iPSC Core Facility, Institute of Stem Cell Research, Helmholtz Center Munich. The VPS45 mutant (Thr224Asn) was generated from the HMGU1 iPS cell line (iPSC#1) and has been described previously (Frey et al., Blood, 2021; PMID: 33512427).
The human renal epithelial cell-derived iPS cell lines 29B5 (iPSC#2, male) and 12C2 (iPSC#5, female) were provided by the lab of W. Enard, Anthropology and Human Genomics, Faculty of Biology, Ludwig-Maximilians-University Munich and generated from urine samples as previously described (Geuder et al., Sci Rep, 2021, PMID: 33568724).
The human PBMC-derived iPS cell line SCTi003-A (iPSC#3, female) was purchased from STEMCELL Technologies (cat. no. 200-0511). The human fibroblast-derived iPS cell line WTC-11 (iPSC#4, male) was purchased via the Coriell Institute (cat. no. GM25256) from the NIGMS Human Genetic Cell Repository.
The mouse MS5 cell line was purchased from DMSZ, German Collection of Microorganisms and Cell Cultures GmbH (cat. no. ACC441).

Authentication

Cell lines were not authenticated.

Mycoplasma contamination

The cell lines used in this study were regularly tested negative for mycoplasm. Testing was performed every three weeks.

Commonly misidentified lines
(See ICLAC register)

The cell lines used in this study are not listed in the ICLAC database of commonly misidentified cell lines.

Animals and other research organisms

Policy information about studies involving animals; ARRIVE guidelines recommended for reporting animal research, and Sex and Gender in Research

Laboratory animals	8-12 week old male and female NSG mice (Charles River) were used for kidney capsule transplantation experiments. 3-4 week old male and female NSG-HLA-DQ8 mice (Jackson Laboratory) were used for HSPC transplantation experiments. Mice were housed under specific pathogen-free conditions in a 12 h light/dark cycle, at 20-22°C temperature, 45-60% humidity and food and water ad libitum.
Wild animals	No wild animals were used in this study.
Reporting on sex	Female and male mice were equally used in this study.
Field-collected samples	No field-collected samples were used in this study.
Ethics oversight	All mice were bred, maintained, examined, and euthanized in accordance with institutional animal care guidelines and ethical animal license protocols approved by the legal authorities. The kidney xenotransplantations were carried out under the animal license number 2022-0.429.375 according to Austrian legislation and approved by the Federal Ministry of Education, Science, and Research of Austria. HSPC xenotransplantations were approved by the Government of Upper Bavaria (Regierung von Oberbayern).

Note that full information on the approval of the study protocol must also be provided in the manuscript.

Flow Cytometry

Plots

Confirm that:

☒ The axis labels state the marker and fluorochrome used (e.g. CD4-FITC).

☒ The axis scales are clearly visible. Include numbers along axes only for bottom left plot of group (a 'group' is an analysis of identical markers).

☒ All plots are contour plots with outliers or pseudocolor plots.

☒ A numerical value for number of cells or percentage (with statistics) is provided.

Methodology

Sample preparation	BMOs were collected and washed once with PBS. Then, organoids were enzymatically dissociated into single cells with 3U/ml Dispase II (Gibco), 2U/ml Liberase (Roche) and 100U DNAse (STEMCELL Technologies) in PBS for 25 min at 37°C. During the incubation period, organoids were mechanically disrupted after 10, 20 and 25 minutes by vigorous pipetting using a P1000 and P200 pipette. After 25 minutes, the dissociation reaction was stopped, and single cells were washed with PBS/2%FBS and collected at 300g for 5 minutes. Cells were resuspended in PBS/2%FBS strained through a 70 µm mesh, counted and incubated for 7 minutes with Fc block (Human True Stain FcX, Biolegend) and subsequently stained with the antibodies listed above and in Supp. Table 1.<br>NSG mice with successful kidney transplants were euthanized and the femur and tibia were harvested. The intact bones were sterilized in 70% EtOH for 60 seconds before the bones were crushed in PBS. The bone marrow was extracted from the crushed bones and passed through a filter. Following filtering, red blood cells were lysed with the BD Pharm Lyse kit (BD Biosciences, 555899) according to the manufacturer's instruction. Properly lysed bone marrow cells were subsequently stained with a mix of eBioscience Fixable Viability Dye eFluor 780 (Invitrogen, 65-0865-14) and CD16/CD32 Fc blocking antibody (BD Biosciences, 553141) in PBS for 20 min at 4°C in the dark. After the first stain, cells were washed with FACS buffer and subsequently stained with antibodies listed in Supp. Table 1.<br>NSG-HLA-DQ8 mice were euthanized 10 weeks and 12 weeks after the transplantation. Bone marrow was harvested by flushing bones with PBS/2%FBS. Blood and bone marrow were stained with antibodies listed in Supp. Table 1 and red blood cells were lysed with the BD FACS Lysing solution (BD Biosciences, 349202). Following two washes with PBS/2%FBS blood and bone marrow cells were filtered through a 35µm strainer cap before analysis.<br>ATOs were submerged in staining buffer (PBS/2% FBS/2mM EDTA), mechanically disintegrated by gentle pipetting and passed through a 50 µm strainer. Cells were subsequently stained with antibodies listed in Supp. Table 1.
Instrument	For Data collection a BD Fortessa LSR flow cytometer (BD Bioscience) was used. Sorting was conducted on a BD FACS Aria cell sorter (BD Biosciences).
Software	FACS DIVA software (BD) v9.0.1 was used to collect flow cytometry data. Flow cytometry data were analysed in FlowJo v10.
Cell population abundance	Post-sort analysis of BMO-derived sorted cells showed a purity of >95%.
Gating strategy	For flow cytometry analysis or sorting, cells were initially gated by FSC-A vs SSC-A for the exclusion of debris. For single cells, samples were further gated by FSC-A vs FSC-H or SSC-A vs SSC-H. Dead cells were excluded by a fixable viability stain. For further gating see detailed gating strategies shown in Ext. Data Fig. 1a, Ext. Data Fig. 5c,e, Fig. 3a, Supp. Fig. 1a, Supp. Fig. 5g, Supp. Fig. 6a, Supp. Fig. 9e-h. Gates were drawn according to FMO controls as indicated in Supp. Fig. 1a, Supp. Fig. 6b.

☒ Tick this box to confirm that a figure exemplifying the gating strategy is provided in the Supplementary Information.
